# Toward Smart Railway Infrastructure Predictive and Optimised Maintenance Through Digital Twin (DT) System

**DOI:** 10.3390/s26082333

**Published:** 2026-04-09

**Authors:** Mahyar Jafar Kazemi, Maria Rashidi, Won-Hee Kang, Mohammad Siahkouhi

**Affiliations:** Centre for Infrastructure Engineering, Western Sydney University, Sydney, NSW 2751, Australia; w.kang@westernsydney.edu.au (W.-H.K.)

**Keywords:** Digital Twin (DT) systems, smart railway infrastructure, predictive and optimised maintenance, Condition-Based Maintenance (CBM), Internet of Things (IoT), Artificial intelligence (AI), cyber-physical systems

## Abstract

**Highlights:**

**What are the main findings?**

- A comprehensive review of sensor-enabled Digital Twin systems for railway infrastructure monitoring.
- Integration of sensing technologies, communication networks, and AI methods for predictive railway maintenance.

**What are the implications of the main findings?**

- Provides a reference framework for developing scalable Digital Twin platforms driven by multi-sensor data.
- Identifies research gaps and future directions for intelligent railway infrastructure and smart maintenance systems.

**Abstract:**

Digital Twin (DT) technology is increasingly recognised as a promising approach for predictive and optimised railway maintenance; however, its current applications remain fragmented and lack systematic evaluation across railway domains. This study aims to critically review DT-enabled monitoring, analysis, and maintenance decision-support systems in railway engineering, while identifying key research gaps and future directions. A DT is defined in this study as an integrated cyber–physical system comprising a physical asset, its virtual representation, and continuous bidirectional data exchange enabling real-time monitoring, prediction, and decision-making. A systematic and transparent review methodology was adopted to select 34 representative peer-reviewed studies published between 2020 and 2025, focusing explicitly on DT applications in railway infrastructure and operations. Among these, a subset of 10 key studies was further analysed in greater depth based on their level of technical implementation, data integration capability, and relevance to predictive maintenance applications, which cover multiple domains, including track systems, rolling stock, bridges, and communication networks. Results show that DT-based approaches can enhance fault detection, enable condition-based and predictive maintenance, and reduce reliance on manual inspections. However, significant limitations remain. Most studies are conceptual or pilot-scale, with limited validation under real operating conditions. Key challenges include a lack of standardisation and interoperability, constraints in real-time scalability, data governance and cybersecurity issues, and insufficient integration of multi-source sensing and advanced analytics. This review provides a structured synthesis of current DT implementations in railway systems and highlights critical gaps that must be addressed to enable scalable, reliable, and fully integrated DT-driven maintenance frameworks.

## 1. Introduction

### 1.1. Global Trend Towards Railway Tracks Monitoring Digitalisation

Railway systems worldwide are rapidly adopting digital technologies to improve track monitoring and maintenance efficiency [1]. Over the past decade, digital monitoring systems have been reported to reduce unplanned track failures by approximately 20–40% and maintenance-related delays by 15–30%, depending on network size and traffic intensity [2,3]. Digitalisation refers to using smart sensors, data networks, and software systems to convert physical railway assets into digital data [4,5]. It allows engineers to collect, process, and analyse large volumes of condition data in near real time. The process has been shown to improve inspection accuracy by 25–50% compared with manual or periodic inspections, while also improving safety and operational performance [6].

In this research, digitalisation is defined as the application of modern digital tools to convert physical railway systems into actionable data for informed decision-making. It links the physical railway infrastructure (tracks, sleepers, ballast, and related components) with the digital domain via sensors, data, and modelling. This connection allows engineers to observe track behaviour continuously, anticipate deterioration trends earlier, and plan maintenance more systematically. Studies report that data-driven maintenance planning can reduce lifecycle maintenance costs by 10–25% and extend asset service life by up to 20% when compared with purely time-based strategies [1,2,5]. In summary, digitalisation marks the shift from traditional inspection-based maintenance to smart, data-driven railway management [7]. A key element in this transformation is the DT, a virtual representation of the real railway system that receives continuous information from sensors installed on tracks and related assets [8,9,10]. DT-based monitoring frameworks have demonstrated 30–60% improvements in early fault detection and 15–35% reductions in corrective maintenance interventions in pilot railway deployments [8,9]. Despite the rapid growth of digitalisation and the increasing use of DT technologies in railway systems, current research remains fragmented. Many studies focus on specific aspects such as sensors, AI-based fault detection, or DT models, but often treat these components separately. As a result, it remains unclear how sensing, communication, data processing, and decision-making function as an integrated system, particularly for predictive maintenance in railway infrastructure. Although DT-based approaches have demonstrated strong potential for improving fault detection and maintenance efficiency, their use in large-scale, real-world railway operations remains limited. This is mainly due to challenges such as interoperability, real-time performance, data quality, and organisational readiness.

To address these issues, this paper aims to provide a clear and system-level understanding of DT-based railway maintenance by focusing on how different technological layers are connected. Unlike many existing review studies, which consider these elements separately, this study takes an integrated view that links physical sensing systems, communication networks, data processing, and DT-based decision-making. By reviewing recent studies, the paper aims to explain current practices, highlight key challenges, and point out possible directions for developing more reliable, scalable, and data-driven railway maintenance systems.

### 1.2. Digitalisation in the Railway Maintenance Context

In the railway maintenance context, digitalisation enables condition-based decision-making by linking inspection, monitoring, and maintenance planning systems. By providing real-time condition data, maintenance teams can prioritise high-risk assets and allocate resources more efficiently, reducing unnecessary interventions across the network [11]. Recent studies report that digitalised maintenance frameworks improve asset availability by 10–25% and reduce unplanned interventions by 20–35% compared with traditional approaches [12,13].

Digital technologies used in railway maintenance can be broadly grouped into two categories: established tools and emerging technologies. Established tools include monitoring sensors, condition-based maintenance (CBM) analytics, and Geographic Information Systems (GIS), which mainly support descriptive and condition-based decision-making. Emerging technologies such as IoT, AI, and DTs enable predictive and optimised maintenance by integrating real-time data with analytical and simulation models [13,14,15]. This classification provides a structured way to assess the level of digital maturity in railway systems [16]. Table 1 summarises these technologies, highlighting their main features, limitations, and contributions to maintenance.

### 1.3. Research Questions and Contributions

This paper aims to understand how DT technology can support smart, predictive, and optimised maintenance in railway systems. It focuses on the interaction between sensing technologies, communication networks, data management, and DT-based decision support for rail infrastructure. Based on a systematic review of 34 papers published between 2020 and 2025, the study addresses the following research questions:

- RQ1: How are DTs currently applied in railway infrastructure for monitoring, diagnosis, and maintenance?
- RQ2: How do sensors, communication technologies, data-processing methods, and AI/ML models interact within DT-based railway maintenance architectures?
- RQ3: What are the main technical, organisational, and standardisation gaps that limit the large-scale use of DTs in railway maintenance?
- RQ4: What future research directions and practical actions are needed to move from pilot DT projects to integrated, real-time smart railway systems?

This review makes three main contributions. First, it provides a unified, end-to-end conceptual framework that links the physical layer (sensors and assets), communication layer (wired and wireless networks), data layer (pre-processing, storage, and analytics), and DT layer (models and decision-making) specifically for railway predictive maintenance (PdM). Second, it synthesises findings from a focused set of 34 representative studies, identified through a structured screening process, across track, rolling stock, bridge, and communication domains, comparing their methods, tools, and levels of DT maturity. Third, it offers a consolidated gap and roadmap analysis, highlighting barriers such as interoperability, data governance, real-time scalability, and skills shortages, and proposes future research directions to support more reliable, sustainable, and standardised DT-based railway maintenance.

### 1.4. Railway Maintenance Challenges, Approaches and Optimisation

#### 1.4.1. Overview of Railway Track Maintenance

**Figure 1:**
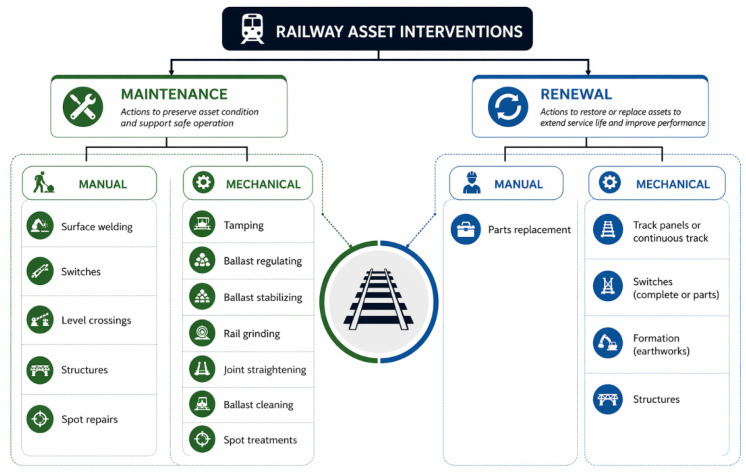
Schematic survey of the maintenance and renewal process.

Track maintenance includes all activities needed to keep railway tracks safe and in good condition at the lowest possible cost [22]. Figure 1 illustrates a simplified overview of the main steps in this process. Maintenance tasks are divided into manual and mechanical activities [22,23]. This division reflects differences in the scale of interventions, the resources required, and the operational impact. Manual work includes local repairs, such as welding worn rails, adjusting switches, fixing drainage, and correcting minor faults identified during inspections [24]. These activities are typically applied over short track sections and are suitable for isolated or low-severity defects, but they are labour-intensive and often require traffic restrictions during execution. Mechanical maintenance uses specialised machines for larger and more repetitive tasks such as ballast tamping, rail grinding, joint straightening, and ballast cleaning to improve drainage and restore track geometry [23]. These interventions are applied over longer track lengths and are designed to restore overall track stiffness and geometry more efficiently than manual methods, thereby extending asset service life [25]. When track components reach the end of their service life, track renewal is conducted. Renewal activities may involve the partial replacement of components in limited sections or the full renewal of longer track segments using dedicated machinery, depending on asset condition, traffic demand, and lifecycle cost considerations [22,26]. Together, maintenance and renewal activities form a hierarchical intervention strategy that balances safety, cost, and long-term performance of the railway track system. The selection between manual, mechanical, and renewal interventions, therefore, depends strongly on timely and accurate condition information, highlighting the importance of data-driven maintenance approaches such as CBM and DT-based decision support [27]. Figure 1 illustrates the hierarchical structure of railway maintenance and renewal, highlighting the progression from manual and mechanical maintenance activities to partial or full renewal of track components depending on asset condition and lifecycle stage [22].

#### 1.4.2. Evolution of Railway Maintenance Strategies

**Figure 2:**
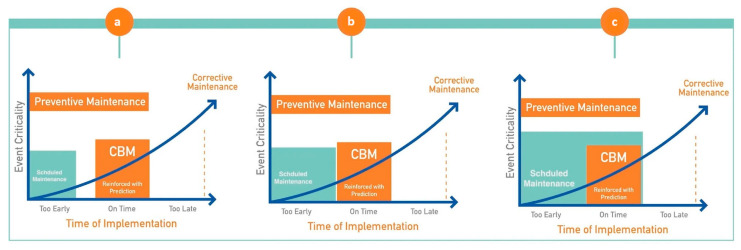
The effects of optimising scheduled maintenance: (**a**) too early scheduled maintenance; (**b**) extended scheduled maintenance; (**c**) on-time scheduled maintenance [43].

Maintenance strategies are commonly grouped into three main categories: reactive, preventive, and predictive [28]. Reactive maintenance is performed after a failure occurs. Although it can restore service, it is costly and unpredictable because faults are not detected in advance [29]. Preventive maintenance follows a time or mileage schedule to reduce failure risk and extend asset life [30,31,32]. However, if not planned carefully, it can result in both early and late interventions [33]. Effective preventive maintenance requires reliable schedules, skilled personnel, and good planning tools [34,35]. The third strategy, predictive or CBM-based maintenance, uses condition information to plan interventions at the most appropriate time [36]. In this research, PdM is treated as part of a wider digital maintenance approach and is discussed in more detail later. In general, PdM and CBM together reduce waste, save resources, and support more sustainable maintenance [36,37]. CBM plays a key role in this process by shifting interventions toward the optimal window shown in Figure 2. Figure 2 illustrates three distinct maintenance-timing scenarios. In scenario (1), maintenance is carried out too early, where preventive actions are applied before significant degradation occurs. While this reduces failure risk, it leads to unnecessary interventions and inefficient use of asset life. Scenario (2) represents delayed maintenance, where interventions are performed too late, after degradation has progressed toward critical levels, increasing the likelihood of corrective maintenance, service disruption, and higher repair costs. Scenario (3) shows the optimal intervention window enabled by condition-based and predictive maintenance, where actions are triggered based on actual asset condition and predicted degradation trends [9]. This scenario balances cost, risk, and asset utilisation by avoiding both premature and late interventions. PdM uses IoT sensors, large datasets, and AI techniques to monitor asset conditions in real time [38,39]. It analyses vibration, temperature, geometry, and other performance indicators to estimate when a failure is likely to occur. According to the International Union of Railways (2024), PdM can improve train reliability by around 15%, reduce maintenance costs by roughly 20%, and cut failures by about 30%. CBM supports this method by continuously observing track and rolling stock conditions to prevent breakdowns [40]. Together, PdM and CBM help the railway sector move from traditional maintenance towards smart, data-driven systems that improve safety, reduce cost, and increase efficiency [41,42].

## 2. Literature Search Method

**Figure 3:**
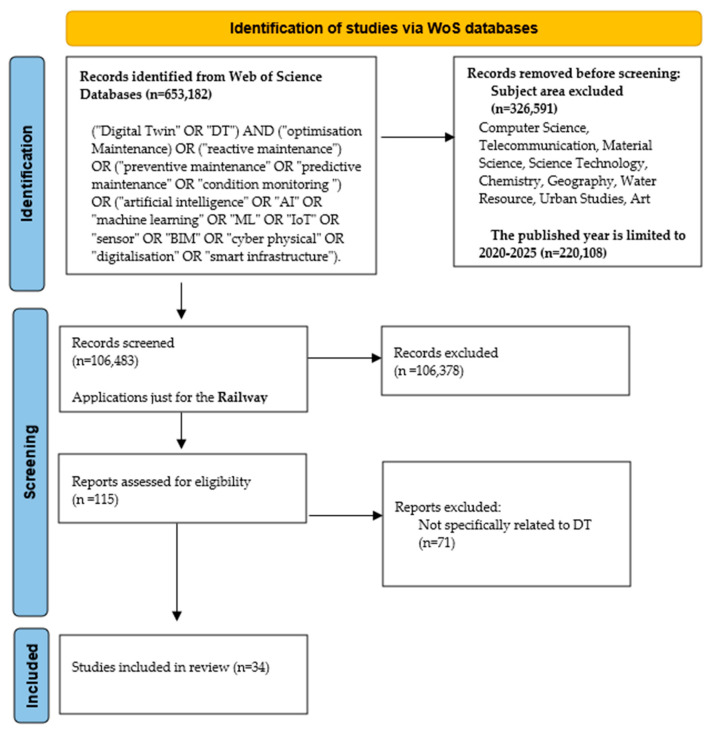
A flowchart illustrates the classification of literature.

This study adopted a systematic and structured literature review methodology to ensure comprehensive coverage, transparency, and reproducibility. Unlike a simple keyword-based search, which may overlook relevant studies due to variations in terminology, publication focus, or disciplinary boundaries, the adopted approach was designed to capture a broad body of relevant research and then progressively narrow it down based on clearly defined criteria. This strategy minimises selection bias and ensures that the final analysis is based on representative and technically relevant studies. The literature search was conducted using the Web of Science (WoS) database, sourcing high-quality peer-reviewed journal articles and conference proceedings from major publishers, including Elsevier (e.g., Automation in Construction, Computers in Industry), IEEE (IEEE Transactions), MDPI (Sensors), ASCE journals, and Taylor & Francis. This ensured the reliability and scientific rigour of the reviewed literature across engineering, transportation, and computer science domains. The research covered the period from January 2020 to January 2025, reflecting the rapid growth of DT research in railway maintenance during recent years. A Boolean search strategy combining multiple keyword groups was applied to identify studies linking DT concepts with railway systems, maintenance strategies, digital monitoring, communication technologies, and data-driven analytics. The Web of Science search was conducted using a Boolean query combining DT concepts with railway infrastructure and maintenance-related terms. The search string was formulated as follows:

(“Digital Twin” OR “DT”) AND (“optimisation Maintenance”) OR (“reactive maintenance”) OR (“preventive maintenance” OR “predictive maintenance” OR “condition monitoring”) OR (“artificial intelligence” OR “AI” OR “machine learning” OR “ML” OR “IoT” OR “sensor” OR “BIM” OR “cyber physical” OR “digitalisation” OR “smart infrastructure”). The initial search returned a large pool of publications. After applying subject-area filters and publication-year restrictions, 220,108 records remained. These records were then screened based on their relevance to railway applications, resulting in 115 papers for further assessment. Titles and abstracts were reviewed to exclude studies not specifically related to DT applications in railway maintenance. A full-text assessment was subsequently conducted to evaluate technical depth, implementation detail, and relevance. This process resulted in 34 studies that met all inclusion criteria. It is important to note that the broader literature on DTs, maintenance, and railway digitalisation extends beyond these selected studies. Among these, a subset of 10 studies was selected for more detailed analysis. However, the selected subset represents studies that explicitly implement, analyse, or validate DT-related architectures, workflows, or decision-support mechanisms in a railway maintenance context. These papers were therefore chosen for in-depth comparative analysis, as they provide sufficient technical detail to examine sensing strategies, communication layers, data-processing pipelines, DT architectures, and validation approaches. The selection process followed a simplified PRISMA-based workflow, as illustrated in Figure 3. While additional studies on DTs, smart infrastructure, maintenance, or railway digitalisation may exist, many do not provide sufficient technical detail or explicit DT-based maintenance implementation to support a consistent comparative analysis. The focus on this subset reflects a deliberate analytical scope rather than an assumption that broader literature is limited to these studies.

Inclusion criteria required studies to be published in English, indexed in Web of Science, fall within the defined time window, and focus on railway applications involving digital monitoring, data-driven analysis, communication-enabled systems, or DT-based maintenance frameworks. Studies were excluded if they addressed railway systems only at a conceptual level, lacked technical implementation detail, or focused on non-maintenance applications. Review papers were excluded from the final analytical set but were used to contextualise trends and identify gaps. A structured data-extraction process was applied to the selected studies to ensure consistency. Extracted information included the railway subsystem under investigation, sensing and data-acquisition methods, communication technologies, data-processing techniques, DT or cyber-physical architecture, and validation type (laboratory, pilot-scale, or real-track deployment). This information was systematically compared to identify recurring patterns, strengths, and limitations across the literature. Several limitations of the review are acknowledged. Restricting the search to Web of Science may exclude relevant studies indexed in other databases or reported in non-academic industry sources. In addition, incomplete reporting of datasets, algorithms, or deployment conditions in some papers limited a deeper technical comparison. Moreover, certain industrial DT implementations are not publicly documented. Despite these constraints, the adopted methodology provides a robust and defensible foundation for analysing recent developments in DT-based railway maintenance and for identifying current capabilities, research gaps, and future research directions.

**Figure 4:**
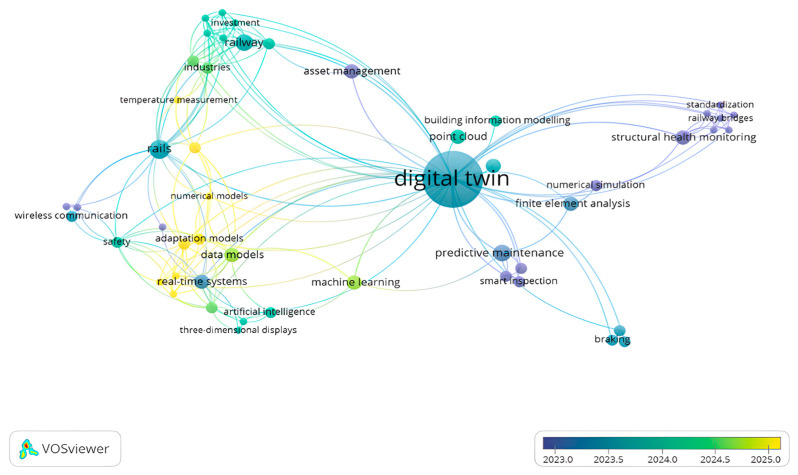
Co-occurring keywords Network.

**Figure 5:**
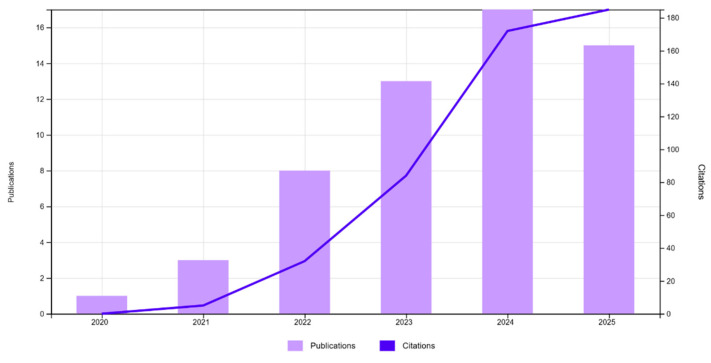
Number of Publications & Citations during 2020 to 2025.

Figure 4 illustrates the co-occurring keyword network for “digital twin” in railway research between 2020 and 2025. The bigger the node, the more often the keyword appears, and the thicker the lines, the stronger the connection between keywords. The colours show the average year of publication. In the early years (blue and purple, around 2023), studies mostly focused on topics like structural health monitoring, railway bridges, standardisation, and numerical simulation. These areas helped build the basic technical foundations of DT technology. In the middle of the network (teal, around 2024), keywords like building information modelling, point cloud, asset management, and railway appear in the centre. These connect modelling tools with how railway systems are managed during their lifecycle. Newer research (green and yellow, closer to 2025) focuses more on data-driven approaches, with keywords such as real-time systems, data models, adaptation models, AI, and ML. This shows a clear shift towards smart, AI-supported DTs. A notable gap revealed by the keyword analysis is the weak integration of temperature-related data into railway DT models, despite the strong influence of thermal effects on rail geometry and stability. This gap indicates the need for future studies that explicitly integrate temperature sensing, weather data, and climate variability into DT-based degradation modelling and maintenance decision-support, particularly under changing environmental conditions. Similarly, the prominence of keywords related to wireless communication highlights future research needs in designing reliable, low-latency data transmission architectures capable of supporting real-time DT updates across large-scale and distributed railway networks. Keywords such as PdM, smart inspection, and braking show how DT technology is moving from basic modelling to real operational applications. Overall, the Figure illustrates how research has evolved from monitoring and simulation to real-time, intelligent DT systems for railway infrastructure. Figure 5 illustrates the number of publications and citations on DT applications in railway engineering from 2020 to 2025, based on 34 papers collected for this study. The number of publications has increased steadily each year, with a clear peak in 2024. Citations also show a strong upward trend, starting slowly but growing sharply from 2023 onward.

## 3. A Conceptual Framework for the Development of Smart Railway

**Figure 6:**
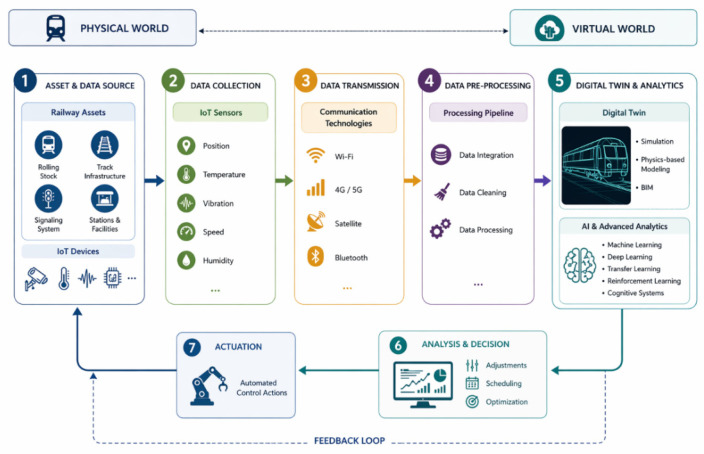
Digital twin for railway.

An integrated approach that links the physical and virtual worlds is required for developing smart railway systems [44]. This allows for real-time monitoring, predictive maintenance (PdM), and data-driven decision-making. As shown in Figure 6, this process begins with gathering data from IoT sensors placed on railway assets [45]. The data is then transmitted and pre-processed to ensure quality and consistency [45]. This stage is critical, as the accuracy and reliability of sensor data directly influence the performance of downstream analytics and DT models [46]. Then, the processed data is put into DT models, where advanced simulation and machine learning (ML) methods are used to analyse, optimise, and schedule maintenance activities [47]. The feedback loop between the real world and the virtual world enables continuous model updating and supports more informed maintenance decisions over time [47,48]. Through this closed-loop interaction, maintenance strategies can be gradually refined based on observed asset behaviour and operational outcomes. This framework outlines the main stages of the DT-based maintenance loop. In the following sections, each stage is analysed in detail, focusing on the key technologies involved, their advantages and limitations, and their effectiveness in supporting different maintenance functions.

### 3.1. Overview of Smart Railway Systems

Smart railways are emerging as a key element of modern transportation, with the global railway digitalisation market projected to exceed USD 150 billion by 2030 [49]. By integrating technologies such as 5G, IoT, AI, and DT systems, smart railways are expected to deliver up to €3 billion in annual savings through predictive and optimised maintenance [50]. In railway engineering, early implementations similar to DTs were simulation models used for diagnostics and asset management [51,52]. The addition of IoT sensors later made it possible to update virtual models in real time, improving PdM and CBM strategies [15]. This framework helps integrate CPS and PdM to improve sustainability and reliability. Two key features are lifecycle integration and real-time interaction through sensor networks [52]. Advanced analytics, cloud computing, ML, and 3D modelling support DT implementations by enabling complex simulations and decision-making [53]. At the foundation of any smart railway or DT system lies the physical layer, where condition data is acquired directly from railway assets through sensing technologies [44,52].

### 3.2. Physical Layer and Sensor Data Acquisition

#### 3.2.1. Physical Components

The physical world includes all real railway assets, such as tracks, sleepers, ballast, bridges, overhead line systems, rolling stock, and signalling devices [38]. In modern railway systems, selected assets and locations are equipped with sensing technologies, including IoT-enabled sensors, to collect operational and structural data [4]. Common sensors include accelerometers and vibration sensors for detecting cracks and dynamic behaviour, temperature sensors for monitoring thermal effects, strain gauges for measuring stresses in rails, ultrasonic sensors for identifying internal defects, LVDT sensors for displacement measurement, and ground-penetrating radar (GPR) or moisture sensors for assessing ballast and subgrade conditions [44,54,55]. These sensors generate continuous condition data that represent the current state of the railway infrastructure and provide the primary input for DT-based analysis and decision-making [55]. In practice, sensor deployment is usually focused on critical zones with higher degradation rates or operational risk, such as switches and crossings, transition zones, and embankments, where detailed monitoring is most beneficial [33].

#### 3.2.2. Sensors

There are two types of condition monitoring for railways based on where the sensors are placed: fixed monitoring and portable (on-board) monitoring [2]. In fixed monitoring, sensors are installed on the railway track permanently so that they can measure the condition of every train that passes through a certain area [8]. This approach provides high-accuracy and continuous data but is limited to localised areas. In contrast, on-board monitoring places sensors on trains, such as on wheels, bogies, or car bodies, allowing the condition of large track sections to be assessed from the train’s perspective [56]. Studies show that on-board accelerometer-based systems can achieve between 96% and 100% accuracy in detecting faults such as corrugation, cracks, and minor geometry irregularities, depending on the defect size and vibration intensity [33,56]. However, their performance decreases for smaller or more subtle defects [33,57]. In addition, real-time data transmission remains a key challenge, as wireless communication may suffer from coverage gaps, leading to delayed or buffered data transfer [58]. Localisation of detected defects is another limitation, since moving sensors do not provide fixed reference points, and GPS-based positioning can introduce errors of several metres [2,8,56,59].

Fixed monitoring systems also face practical limitations. Their deployment across large networks is constrained by power requirements, maintenance needs, and physical vulnerability to environmental conditions such as vibration, ballast impact, and weather exposure [8,60]. As a result, their operational lifespan can be limited without proper protection. In addition, installation is time-consuming, and data coverage remains restricted to specific locations, which limits their effectiveness for network-wide assessment [61,62]. Because of these challenges, fixed monitoring is typically applied only in critical zones such as switches and crossings, bridges, transition zones, or high-speed lines, not across the entire network. In these critical zones, however, fixed sensors have demonstrated substantial benefit [23,63]. For example, turnout strain-monitoring systems have enabled the prediction of maintenance needs with reported improvements of 20–35% in scheduling accuracy, because strain patterns correlate closely with axle load and deterioration behaviour [44,64,65]. Laser-based displacement sensors used in turnout panels have also shown high measurement accuracy (above 95% repeatability), but operational deployment remains limited due to environmental sensitivity and setup complexity [66,67].

Overall, fixed monitoring offers high-precision, continuous data at selected locations, while on-board monitoring provides broader spatial coverage with reduced localisation accuracy. Combining these approaches can improve overall condition assessment, although challenges related to communication reliability, cost, and scalability remain. Table 2 provides a comparative overview of commonly used sensor technologies in railway monitoring, including their typical applications, advantages, limitations, and indicative measurement sensitivity.

### 3.3. Cyber-Physical Connectivity and Communication

#### 3.3.1. Data Transmission and Cyber-Physical Connectivity

A communication layer acts as the bridge between the physical and virtual domains to transfer collected data within a railway DT system [52]. Data transmission technologies can generally be divided into wired and wireless systems, including fibre-optic networks, Wi-Fi, LoRaWAN, 4G/5G, and satellite communication, each offering distinct advantages depending on the application environment and network requirements [75]. In practice, fibre-optic networks remain the most reliable option for railway operations, with reported data-loss rates typically below 1–2% in safety-critical environments where uninterrupted connectivity is essential [75,76]. Wireless communication systems provide greater flexibility for mobile assets and trackside deployments, but show more variable performance under real railway conditions [77]. Field measurements indicate packet-loss levels ranging from 5% to 18% in tunnels, rural areas, and deep cuttings, depending on radio propagation, network density, and environmental constraints [77,78,79]. These performance differences explain why modern railway systems increasingly adopt hybrid communication architectures, combining wired backbones with wireless access technologies rather than relying on a single transmission method [21,52]. In many railway applications, edge computing devices are installed near the track or onboard rolling stock to perform local data processing before transmission to central or cloud-based platforms [80,81]. This approach reduces communication latency and bandwidth requirements, which is particularly important for real-time and near-real-time PdM applications [52]. Field and pilot deployments indicate that edge processing can reduce raw data volume by up to 70% before transmission and improve end-to-end system responsiveness by approximately 30–45%, especially when handling high-frequency vibration, strain, or environmental monitoring data [52,78,79,81]. These gains have motivated the deployment of distributed edge nodes across modern railway networks as sensor density and data rates continue to increase.

**Figure 7:**
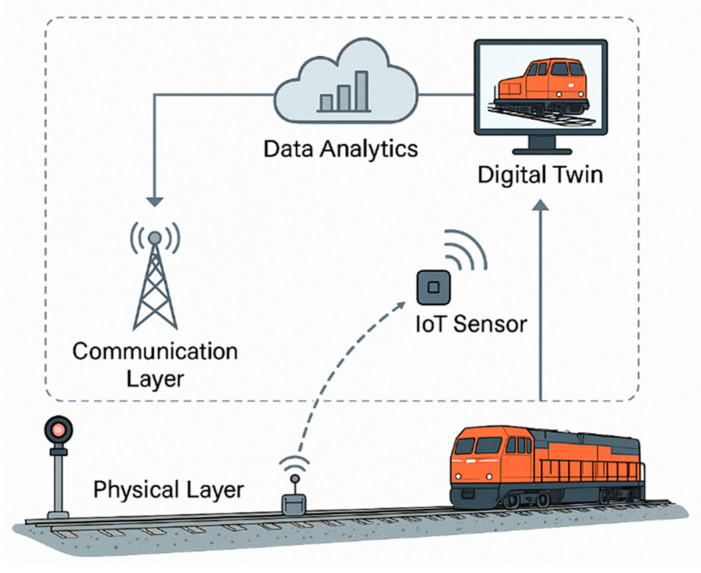
A conceptual illustration of IoT-enabled cyber-physical connectivity in smart railway systems.

Cybersecurity has become a critical requirement in railway communication infrastructures [82]. To protect operational data from tampering, unauthorised access, or service disruption, DT platforms increasingly incorporate security mechanisms such as encrypted communication channels, secure authentication, blockchain-based data logging, and intrusion detection systems (IDS) [82]. Recent reports indicate that European transport networks experienced an increase of more than 20% in recorded cyber incidents between 2020 and 2023, including attempts to interfere with signalling and communication systems [5,46,70]. These trends highlight the necessity for secure, resilient communication layers to ensure the safe and reliable operation of cyber-physical railway systems [21]. Figure 7 illustrates how condition data collected at the physical layer are transmitted through wired and wireless communication infrastructures to data analytics platforms, where they are processed and integrated into the DT. This representation emphasises the role of the communication layer as the operational link that enables continuous interaction between physical railway assets and their virtual counterparts.

#### 3.3.2. The Role of the Internet of Things (IoT) in Railway Infrastructure Maintenance and Management

For digital technologies to be effective in railway systems, sensing is essential for collecting condition data, while connectivity enables communication between sensors, actuators, computing devices, and control platforms [39]. IoT provides this connectivity by linking sensors installed on locomotives, tracks, and stations to central data platforms, thereby supporting safer, more reliable, and more efficient railway operations [39]. IoT technologies have become an important enabler for infrastructure maintenance and asset management by allowing continuous monitoring rather than periodic inspections [16]. Several case studies report that IoT-based monitoring improves inspection efficiency by approximately 25–40% compared with traditional manual inspection cycles, largely because continuous data availability shortens fault detection and response times [68,83]. Although IoT systems do not perform prediction on their own, they provide the continuous data streams required by analytical and AI-based tools to support predictive decision-making [68,83]. Through this data-driven process, IoT-enabled monitoring has been shown to improve network availability, operational efficiency, and asset service life [83,84]. In some operational trials, integrating IoT sensing with basic analytics resulted in up to 18% reductions in unplanned maintenance events, demonstrating the practical value of real-time condition data [83,84].

Track condition monitoring is one of the primary applications of IoT in railways. Sensors are used to measure vibration, geometry, forces, and environmental parameters to identify early repair needs [4,84]. On-board IoT vibration monitoring systems have demonstrated detection accuracies between 96% and 100% for geometry irregularities and corrugation under favourable conditions, depending on defect severity and signal quality [85,86,87]. IoT is also used for asset tracking and operational monitoring of trains and equipment [88,89]. Trials using RFID- and GPS-enabled IoT solutions have achieved localisation accuracies exceeding 98% in controlled environments, although accuracy typically degrades in tunnels or dense urban areas due to signal attenuation and multipath effects [87,88].

Despite these benefits, most IoT-based railway studies remain limited to small-scale pilots or single subsystems [14]. Across the reviewed literature, more than 70% of IoT railway implementations were evaluated on restricted testbeds or individual routes, with only a limited number demonstrating network-level scalability [39]. Large, mixed-traffic railway networks introduce additional challenges, including heterogeneous device fleets, legacy signalling systems, and strict safety and reliability requirements. Packet loss, device interoperability, and cybersecurity constraints remain persistent barriers in IoT-enabled railway systems [90,91]. As a result, IoT adoption across railway networks remains uneven, and many operators continue to rely on manual inspections or simple threshold-based alarms rather than fully integrated IoT–DT platforms [4,92]. Importantly, several studies report that IoT deployment alone does not necessarily lead to improved maintenance planning when data integration, analytics capability, or communication reliability are insufficient [69,78]. This limitation reinforces the role of DT frameworks in integrating IoT data with analytics, simulation, and decision-support functions to enable more effective and scalable maintenance strategies [21,52]. Table 3 summarises the main cybersecurity, technological, social, and information system challenges associated with the use of IoT in railway maintenance and asset management.

##### Wireless Communication in the Railway System

**Figure 8:**
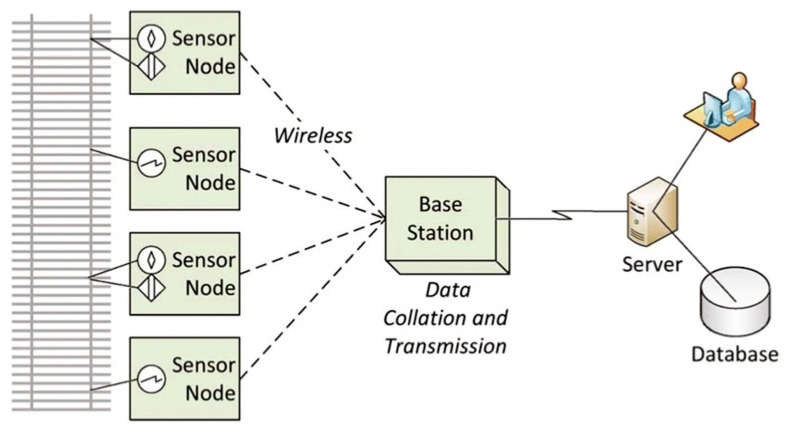
Wireless Sensor Network Configuration for Railway Condition Monitoring.

Wireless communication is an essential component of modern railway monitoring and control systems [99]. It enables sensor data to be transmitted without physical cabling and supports real-time or near-real-time data exchange from sensors deployed across tracks, rolling stock, bridges, and stations [30,33]. Operational studies indicate that wireless transmission success rates typically range between 85% and 98%, depending on environmental conditions, with performance degrading in tunnels, deep cuttings, and dense urban areas due to signal attenuation and multipath effects [77,78,84]. Wireless systems are widely adopted in railway environments because installing and maintaining wired connections can be difficult, costly, or unsafe in dynamic operating conditions such as long track sections, tunnels, bridges, and areas with continuous train movement. In these locations, cables are exposed to vibration, weather, and mechanical damage, making wireless solutions more practical [21,52]. Technologies such as LoRaWAN, Wi-Fi, 4G/5G cellular networks, ZigBee, and satellite communication are commonly used to transmit sensor data to central or cloud-based platforms for processing and analysis [13,100]. Reliable wireless data transfer supports PdM by enabling earlier fault detection, improved operational safety, and extended asset service life [84]. Figure 8 illustrates a typical wireless sensor network configuration for railway condition monitoring, where distributed sensor nodes transmit data to a base station that aggregates and forwards information to backend servers and databases. Such architectures are commonly employed in both trackside and on-board monitoring systems to support scalable data collection.

LoRaWAN is a widely adopted low-power wide-area network (LPWAN) communication protocol that leverages LoRa (Long Range) wireless technology to transmit data from distributed IoT sensors to remote platforms [36]. Owing to its low power consumption, long communication range, and relatively low deployment cost, LoRaWAN is well-suited for non–real-time railway sensing applications such as long-range infrastructure monitoring, remote asset tracking, and condition reporting in low-traffic corridors. Pilot studies report message delivery success rates above 95% in short-range scenarios and approximately 80–90% in mixed operating environments, making LoRaWAN suitable for diagnostic and monitoring tasks where low data rates are acceptable [101]. By contrast, 5G and emerging 5G-R/FRMCS systems are intended for low-latency and high-throughput applications such as real-time monitoring of trains, signalling systems, and large numbers of IoT devices [77,102,103]. Other wireless technologies occupy intermediate positions in the performance–coverage trade-off. Wi-Fi-based monitoring systems deployed on inspection vehicles or within stations have achieved transmission speeds of 30–90 Mbps in open-track tests, but their short-range limits applicability in rural environments [104]. ZigBee offers very low power consumption and mesh networking capabilities, but is typically restricted to short ranges (often <100 m), making it unsuitable for high-speed or long-distance railway applications. Satellite communication provides global coverage and is valuable for remote or isolated rail corridors; however, high latency levels (often >500 ms) and operational costs limit its suitability for time-critical monitoring and control tasks [84,105].

Overall, no single wireless technology can satisfy all railway communication requirements across diverse operating environments. As a result, many railway systems adopt hybrid communication architectures that combine wired backbones with local wireless access technologies [52,84]. From a DT perspective, the choice of wireless technology directly affects data timeliness, reliability, and scalability, and therefore influences the effectiveness of DT-based monitoring, analytics, and maintenance decision support.

##### Wired Communication in the Railway System

Wired communication systems have long formed the backbone of railway operations for monitoring, signalling, and control. These systems transmit data between trackside equipment and control centres using copper cables, optical fibres, or track circuits. Wired connections remain the preferred choice for safety-critical functions such as signalling, train detection, and data acquisition from fixed sensors installed on tracks or bridges [106].

Field observations indicate that optical-fibre–based signalling networks typically operate with data-loss rates below 0.1% and latency levels under 5 ms, explaining their continued use as the primary communication backbone in modern railways [106]. Wired systems are preferred for these applications because they offer high reliability, very low latency, and strong resistance to electromagnetic interference. Latency performance varies across wired media: optical fibre generally exhibits 1–5 ms latency, whereas copper cabling often shows 5–20 ms due to higher electrical resistance and electromagnetic sensitivity [107]. In addition, monitoring studies report that physical tampering or electromagnetic disturbance accounts for less than 1% of communication failures in wired systems, which is substantially lower than disturbance rates observed in wireless environments near traction equipment [108].

Despite these advantages, wired systems face practical limitations. Installation and maintenance over long distances can be expensive, and network upgrades often require extensive physical work. Maintenance is also more difficult in tunnels, bridges, and protected corridors, where replacing buried or shielded cables is costly. These factors reduce the flexibility of wired systems for large-scale deployment of modern IoT devices, which often require faster installation and wider spatial coverage. For this reason, many railway networks now adopt hybrid architectures in which wired communication supports safety-critical signalling and control, while wireless technologies provide flexible access for distributed, non-critical monitoring applications [107]. From a DT perspective, this approach allows time-critical functions to rely on the stability of wired communication without limiting the scalability of wider monitoring systems. Table 4 summarises the main wired communication technologies used in railway monitoring and maintenance, highlighting their typical applications, strengths, and limitations.

#### 3.3.3. Cyber-Physical Connectivity

**Figure 9:**
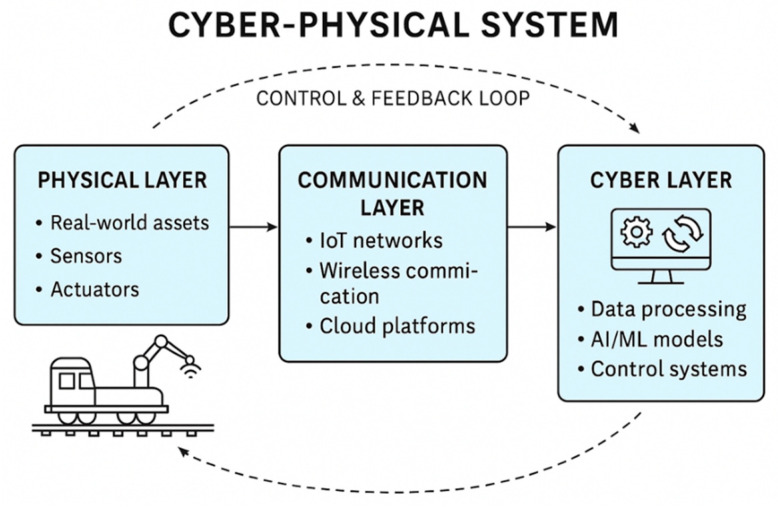
CPS structure showing physical, communication, and cyber layers with a closed-loop feedback system.

Cyber-physical systems (CPS) provide the functional structure that connects the physical railway environment with its digital representation. In a CPS, data are collected from physical assets, transmitted through communication networks, interpreted within the cyber layer, and then used to support responses or actions in the physical system [119]. This continuous exchange of information and control signals across the physical, communication, and cyber layers forms the closed-loop structure of a CPS, as illustrated in Figure 9. At the connectivity level, CPS focuses on the reliable acquisition of condition data from sensors, IoT devices, and railway equipment. Reported railway CPS studies associate this improved data availability with reductions of approximately 20–30% in inspection-related effort, as asset information can be accessed more frequently without requiring on-site inspection [120,121]. At the data-to-information conversion level, structured data flow across CPS layers has been linked to 15–25% reductions in decision-response time, as information becomes more accessible and consistent across systems [52,122].

The cyber and cognition levels support analysis and decision-making. The cyber layer models system behaviour, while the cognition layer interprets analytical results to support maintenance and operational actions. In reported railway applications, this has been associated with reductions of about 10–15% in unplanned maintenance interventions [21]. The configuration level then adapts responses based on feedback from both the physical and cyber layers, with some studies reporting 5–12% improvements in operational stability and asset availability compared with static or manually driven approaches [20,123]. Together, these CPS levels create an adaptive and data-driven link between the physical railway system and its digital counterpart. The quantitative values reported here should be understood as indicative trends from pilot studies rather than universal performance guarantees.

### 3.4. Data Management and Analytics

#### 3.4.1. Data Pre-Processing

Poor data accuracy can seriously affect the performance of a DT, as every layer of sensing, communication, data processing, AI modelling, and decision-making depends on reliable input. When incoming data are incomplete or erroneous, virtual models may update incorrectly, AI algorithms may learn from distorted patterns, and the system can generate unreliable predictions or maintenance decisions. Accurate and consistent data are therefore essential for DT-based applications [124]. Grieves and Vickers (2016) further emphasise that the quality of sensor data acquired from physical assets directly determines the fidelity of the digital representation [9]. Several railway case studies report that improvements in raw data quality alone can increase downstream prediction accuracy by approximately 10–20%, even before the application of advanced AI models, highlighting pre-processing as a critical stage for DT reliability [9,46].

Before analysing vibration and acoustic data, frequency-domain techniques are commonly applied to extract meaningful patterns from raw time-series signals. The Discrete Fourier Transform (DFT) converts a discrete time-domain signal *x*(*n*) of length *N* into its frequency-domain representation *X*(*k*) defined as [125]:

(1) $$X\left(k\right)=\sum_{n=0}^{N-1}x\left(n\right)e^{-j\frac{2\pi kn}{N}}\;k=0,1,\ldots ,N-1$$

where $x\left(n\right)$ is the discrete time-domain signal, $X\left(k\right)$ is the complex frequency-domain representation (spectrum) of the signal, “*N*” is the total number of samples, *n* is the time index, and “*k*” is the frequency index. The Fast Fourier Transform (FFT) is a fast way to calculate the DFT that uses less computer power. In railway monitoring applications, FFT-based analysis has demonstrated detection accuracies typically in the range of 85–95% for repetitive defects such as corrugation and periodic geometry irregularities, provided that the signal remains stationary [87,126]. However, railway signals are often non-stationary, as impacts from rail joints, wheel flats, sleeper defects, fastening failures, and ballast irregularities introduce short-duration and time-varying events that alter spectral characteristics over time. Under such conditions, FFT alone is insufficient to fully capture transient behaviour. To address non-stationary signal characteristics, advanced time–frequency methods are widely employed. The Short-Time Fourier Transform (STFT) analyses signals within sliding windows, enabling simultaneous observation of temporal and spectral variations [125]:

(2) $$STFT\left\{x\left(t\right)\right\}\left(t,\omega \right)=\int_{-\infty }^{+\infty }x\left(\tau \right)w\left(t-\tau \right)e^{-j\omega \tau }d\tau$$

where (⋅) ω is a window function controlling the trade-off between time and frequency resolution. Wavelet Transform (WT) techniques provide even higher adaptability for transient event detection. The Continuous Wavelet Transform (CWT) is defined as [125]:

(3) $$W\left(a,b\right)=\frac{1}{\sqrt{\left|a\right|}}\int_{\left\{-\infty \right\}}^{+\infty }x\left(t\right)\Psi^{*}\left(\frac{t-b}{a}\right)dt$$

where “*a*” denotes the scale parameter, “*b*” the translation parameter, *ψ*(*t*) the mother wavelet, and $(.)\Psi^{*}$ is the complex conjugate. Railway studies consistently show that wavelet-based analysis improves the detection of short-duration events, such as wheel flats and sleeper cracks, by approximately 22–30% compared with FFT-only approaches, particularly when analysing impact-dominated signals [38,127,128]. These methods, therefore, form an essential component of robust pre-processing pipelines for railway DT applications.

ML techniques increasingly support the pre-processing stage by enhancing feature extraction, noise reduction, and early fault pattern recognition. Convolutional Neural Networks (CNNs) have proven effective for analysing vibration and acoustic spectrograms, while recurrent models such as Long Short-Term Memory (LSTM) and Gated Recurrent Unit (GRU) networks capture temporal dependencies in time-series data. Autoencoder architectures are commonly used for denoising and anomaly detection, whereas classical models such as Random Forests and Support Vector Machines (SVMs) are applied to classify hand-engineered features. For example, Gao et al. (2022) demonstrated that a CNN–Autoencoder pipeline improved acoustic signal clarity by approximately 18–25% near rail joints, thereby enhancing data interpretability for subsequent modelling stages [76]. Similarly, GRU-based denoising approaches have been reported to reduce background railway noise by around 20%, making it easier to find weak defect signatures [129].

**Figure 10:**
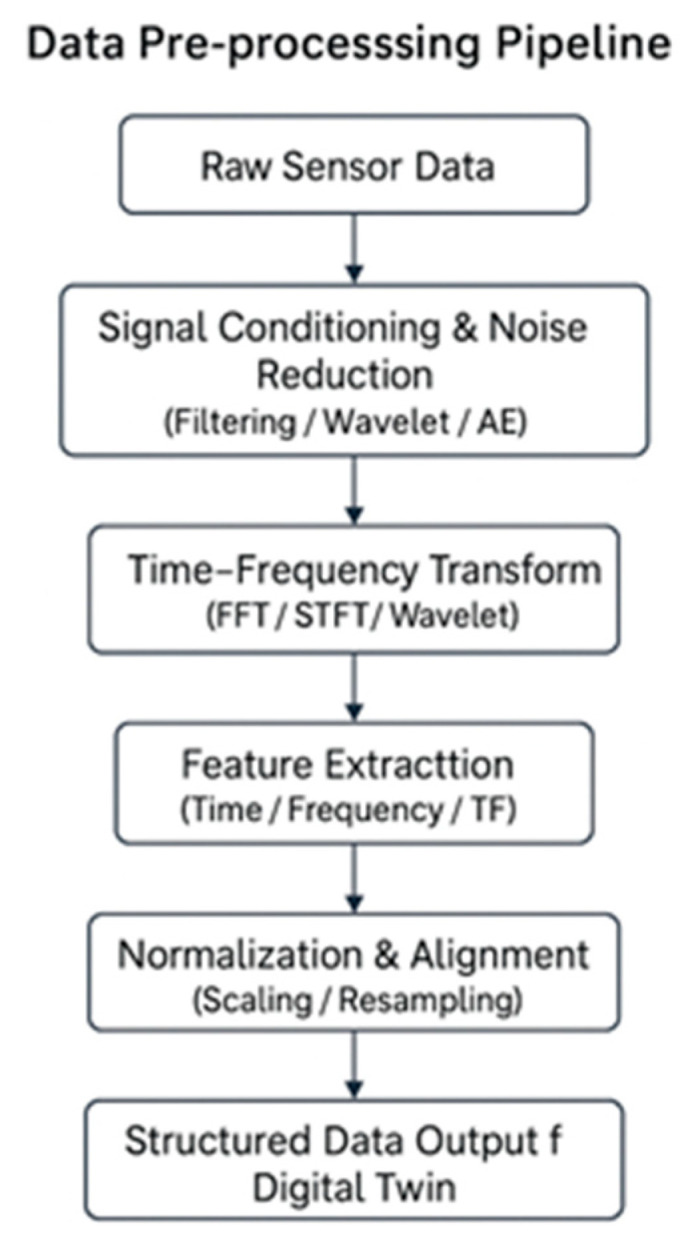
Data pre-processing pipeline transforming raw sensor signals into structured inputs for Digital Twin analytics through noise reduction, time–frequency analysis, feature extraction, and normalisation.

A range of software tools supports these pre-processing methods. MATLAB (R2023a) provides dedicated libraries such as the Signal Processing Toolbox and Wavelet Toolbox for filtering, spectral analysis, and time–frequency decomposition [130,131]. Python-based environments (version 3.10), including NumPy, SciPy, Pandas, PyWavelets, and Scikit-learn, are widely used for large-scale time-series processing and feature extraction [111,132]. Many IoT-oriented railway studies employ stream-processing platforms such as Apache NiFi to manage real-time data flows [111]. While earlier works relied on manual or spreadsheet-based processing, such approaches do not scale for DT or IoT applications. More recent studies increasingly adopt cloud platforms, including Microsoft Azure IoT Hub, AWS IoT Core, and Google Cloud Dataflow, to support large-scale data handling [112]. However, cloud-based processing can introduce latency and security concerns, leading many railway DT implementations to adopt hybrid edge cloud workflows. Reported deployments indicate that such hybrid architectures can reduce total processing time by up to 35% for high-frequency vibration data streams, making them well-suited for DT-based monitoring and analytics [21,77,133]. Figure 10 illustrates a typical data pre-processing pipeline that transforms raw sensor signals into structured inputs for DT analytics through noise reduction, time–frequency analysis, feature extraction, and normalisation. Table 5 summarises the main pre-processing methods, their purposes, advantages, limitations, commonly used software tools, and representative studies.

#### 3.4.2. Analysis, Prediction, and Optimisation

Once data enter the DT, analytical models transform the information into insights that support condition assessment and maintenance decision-making. At this stage, DT-based analytics focus on three core functions: detecting abnormal behaviour, predicting degradation trends, and identifying efficient maintenance actions. Across reported railway studies, the application of ML and deep learning (DL) at this analytical stage has been associated with improvements in diagnostic accuracy of approximately 10–35% compared with rule-based or threshold-driven approaches, depending on asset type, data quality, and operating conditions [21,52,134]. For anomaly detection, traditional ML models such as Random Forests, SVM, and K-Nearest Neighbour (KNN) are widely applied because they can identify patterns that deviate from normal operating behaviour. In railway monitoring applications, these methods have demonstrated detection accuracies typically in the range of 85–97%, enabling the identification of abnormal vibration signatures, loading patterns, and thermal deviations that may indicate emerging faults [69,135]. The quality and structure of the input data strongly influence their performance, and they continue to serve as structures of the input data, but they remain effective baseline approaches for early fault detection.

Failure prediction generally requires more advanced models, as railway degradation processes are nonlinear and evolve over time. CNNs perform well when sensor data can be represented in spatial forms such as vibration spectrograms, ultrasonic images, or surface-condition maps. CNN-based railway studies commonly report classification accuracies in the range of 92–98% for surface-level and geometry-related defects [113,115]. Recurrent models, including LSTM networks, are more suitable for sequential data and have demonstrated 15–25% lower prediction error compared with traditional time-series methods when estimating degradation trends and remaining useful life (RUL) [60]. No single ML or DL architecture can handle all railway data types, as each model is designed to exploit specific data structures, spatial, temporal, or statistical, making appropriate model selection critical for reliable DT analytics.

**Figure 11:**
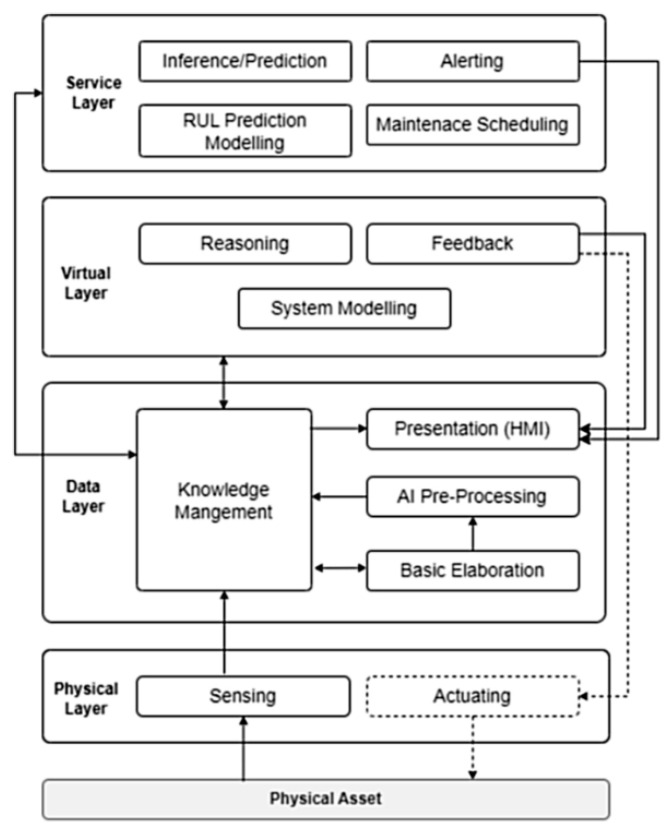
Layered Digital Twin architecture for railway maintenance, illustrating the flow from sensing and data analytics to inference, RUL prediction, and maintenance decision support. Feedback to the physical system represents maintenance planning and human-in-the-loop intervention rather than automated control [1].

Once defects and degradation trends are identified, optimisation techniques are applied to determine suitable maintenance responses. Genetic Algorithms (GA) are commonly used to explore large solution spaces and identify near-global optima for long-term maintenance planning. Reinforcement Learning (RL) methods enable adaptive decision-making by updating policies as new operational data becomes available. Multi-Objective Optimisation (MOO) frameworks support the balancing of competing objectives such as cost, safety, reliability, and downtime [80]. In reported railway case studies, optimisation-driven DT implementations have been associated with 8–15% reductions in maintenance cost and 10–20% reductions in downtime, primarily by avoiding unnecessary interventions and improving maintenance timing [80]. For practical implementation, tools such as MATLAB Simulink and Python-based libraries, including scikit-learn, TensorFlow, Keras, and PyTorch, are widely used to develop, train, and validate prediction and optimisation models [10]. In large-scale scheduling and resource-allocation problems, optimisation solvers such as IBM CPLEX are sometimes employed. These platforms allow researchers and practitioners to test alternative maintenance strategies, evaluate prediction performance, and analyse decision outcomes under controlled scenarios. Despite the strong performance reported for CNN-, LSTM-, and RL-based approaches, many railway DT studies continue to rely on limited or proprietary datasets. When training data are scarce, DL model accuracy can decrease by 10–25% due to overfitting, and results often fail to generalise across different networks [117,134]. The lack of shared benchmark datasets also limits the objective comparison of models. More transparent reporting, larger multi-operator datasets, and open benchmarking efforts are therefore required to strengthen confidence in analytical DT systems and support their wider adoption in operational railway environments. In Figure 11, the feedback from the DT to the physical layer is limited to maintenance decision support and planning, with actions implemented through human operators rather than direct automated control [1]. Across the reviewed architectures, sensors provide continuous condition data that is transmitted through wired or wireless communication layers, pre-processed to ensure quality, and analysed using ML and DL models within the DT. The outputs of these analytical models are then used to support optimisation and maintenance decision-making, illustrating a clear interaction chain from sensing and communication to data processing, analytics, and DT-based decision support.

### 3.5. Digital Twin and Closed-Loop Control

#### 3.5.1. Digital Twin in Railway

**Figure 12:**
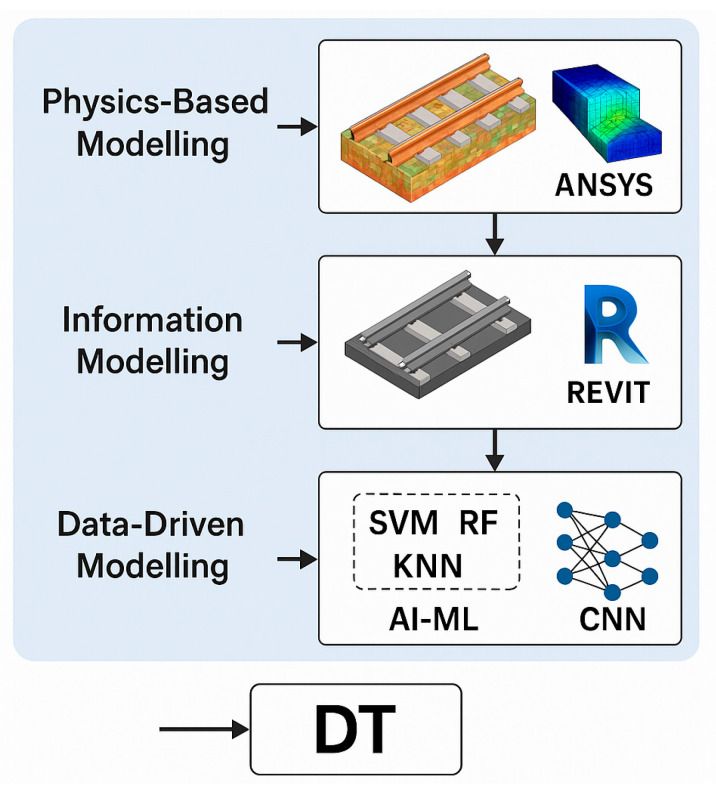
Three-layer modelling architecture of a Digital Twin (DT) for railway systems, consisting of physics-based modelling (e.g., FE simulations in ANSYS), information modelling (e.g., BIM platforms such as Revit), and data-driven modelling using machine learning and deep learning algorithms. Each layer contributes complementary insights that are integrated into the DT for prediction, analysis, and real-time decision support.

The DT is a virtual representation of the real railway system (e.g., an object, system, network, or process) utilising real-time data, simulation models, and AI algorithms to replicate and predict asset behaviour [10]. It serves as the computational core of the cyber-physical system, giving it a dynamic, data-driven model that changes along with the real infrastructure. DT generally consists of three modelling layers, each contributing a different aspect of system understanding and prediction [116,118]. The DT architecture is summarised in Figure 12.

**Figure 13:**
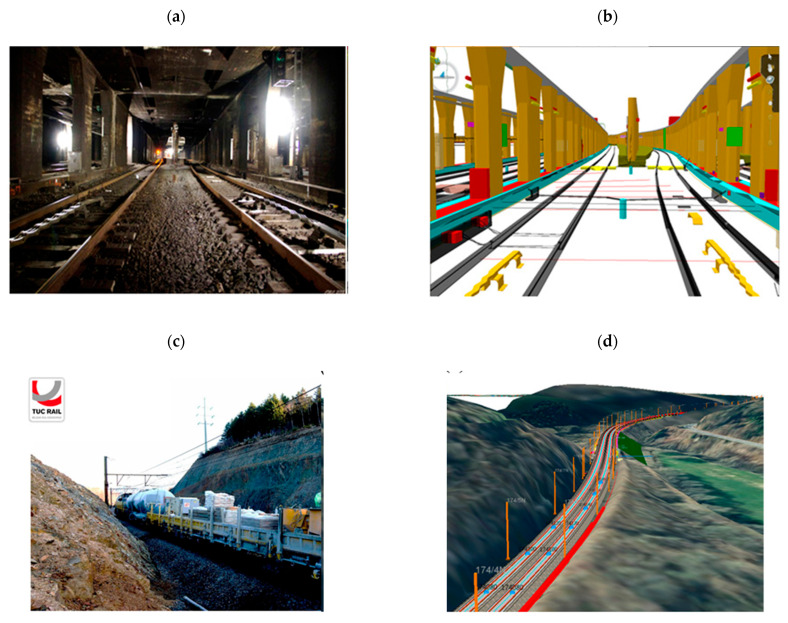
(**a**) Examples of rail infrastructure projects of TUC RAIL, (**b**) with the corresponding BIM model, (**c**) Renovation of the North-South connection, Brussels; (**d**) with the corresponding BIM model [137].

The first layer, physics-based modelling, uses engineering and numerical simulations to reproduce railway assets’ structural and mechanical behaviour. ANSYS, Abaqus, and OpenSees are popular tools for analysing stress distribution, deformation, fatigue life, and temperature impacts in railways, sleepers, and bridges [136]. A finite element model is helpful because it accurately reproduces the geometry of the structure, indicates where the boundary conditions are, and gives a better understanding of local deformations. However, the main drawback is that it takes a long time to run. The second layer, information modelling, focuses on the geometric and semantic representation of assets using BIM systems like Autodesk Revit, Bentley OpenRail, and Navisworks. BIM’s interface with DT systems allows visualisation of track geometry, ballast condition, and sleeper performance, facilitating data-driven maintenance and lifecycle management [51,137]. Figure 13 provides examples of railway projects and their corresponding BIM representations. BIM can also support lifecycle assessment (LCA) by linking maintenance practices such as ballast cleaning, sleeper replacement, and track geometry correction with energy use, carbon emissions, and waste generation [137]. For instance, Kaewunruen et al. [138] showed that digitally supported optimised ballast and sleeper replacement schedules decreased maintenance frequency by as much as 15%, reducing related CO_2_ emissions. Similarly, Bentley Systems [15] reported that PdM powered by DT and CPS in the HS2 project could cut carbon emissions by 3.2 million tonnes over 60 years. The third layer, data-driven modelling, uses AI and ML techniques to interpret sensor data and predict system behaviour. SVM, Random Forests, and KNN are popular algorithms for pattern identification and anomaly detection. Advanced DL architectures, such as CNNs and LSTM networks, are used to analyse spatial and temporal features in large-scale time-series data generated by IoT sensors [60]. CNNs are particularly good at identifying defects in visual or vibration-based data, while LSTMs capture sequential dependencies to estimate degradation patterns and remaining useful life.

In DT–based railway maintenance, selecting the right analytical model depends strongly on the type of data and the behaviour of the underlying physical system. Railway sensor data vary widely: some are spectral, some are spatial, and many evolve temporally, so each model captures a different aspect of track or rolling-stock degradation [52,134]. Classical models such as SVM remain valuable when the dataset is relatively small or when the distinction between normal and abnormal conditions is sharp. In practice, railway applications often use SVM with RBF or polynomial kernels, tuned through penalty parameters such as C and γ, because these kernels are effective at separating subtle defect signatures in early-stage vibration or temperature changes where labelled samples are scarce [69]. Random Forests (RF) are preferred when data comes from multiple heterogeneous sensors. They can assess which variables contribute most to a fault and remain robust even when some measurements are missing or noisy. Typical RF configurations in railway studies include 100–500 trees with moderate maximum depth, which help stabilise predictions in mixed-traffic environments without requiring highly engineered features [69].

**Figure 14:**
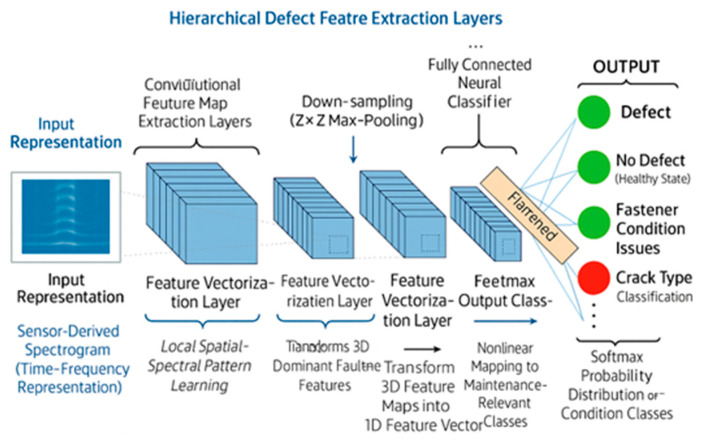
CNN architecture for railway defect classification using spectrogram inputs.

DL models play a different role. CNNs are well-suited to railway data once the signals are transformed into spatial representations such as spectrograms, wavelet scalograms, ultrasonic B-scans, or rail-surface images. These formats reveal localised energy patterns associated with cracks, wheel flats, fastener issues, or impact events [113,139]. Figure 14 illustrates a typical CNN architecture adapted for railway defect detection, showing how convolutional layers extract hierarchical spatial–spectral features from sensor-derived spectrograms before mapping them to maintenance-relevant output classes. Railway implementations typically use two or three convolutional layers with 3 × 3 or 5 × 5 kernels, batch normalisation, and dropout between 0.3 and 0.5 to prevent overfitting, enabling the network to learn defect-specific features that are difficult to engineer manually. In contrast, degradation processes that unfold over time require models capable of capturing long-range temporal dependencies. LSTM networks are particularly effective for this task because they learn how condition indicators evolve across repeated loading cycles and environmental transitions. LSTMs used in DT prediction often contain three to five hidden layers, sequence lengths of 50–200 time steps, and approximately 10–20 training epochs, which together allow them to predict emerging failures and remaining useful life (RUL) with lower error rates than classical time-series models [60,139]. By integrating these different modelling approaches, the DT gains a comprehensive analytical capability: SVM and RF provide interpretable baselines for heterogeneous or limited datasets, CNNs extract spatial-frequency signatures of defects from transformed sensor images, and LSTMs capture the temporal evolution of degradation. This multi-model perspective enables the DT to reflect both the complexity of railway data and the distinct mechanisms through which faults develop, ultimately supporting a richer and more reliable maintenance decision process [52,134].

Table 6 provides a comparison of commonly used ML and DL models in railway DT applications, showing how each model aligns with different data types and maintenance objectives, and Table 7 provides a comparative summary of ML and DL models used in railway DT applications, highlighting their suitable data types, key strengths, limitations, and typical performance reported in the literature.

#### 3.5.2. Alternatives to Digital Twin

**Figure 15:**
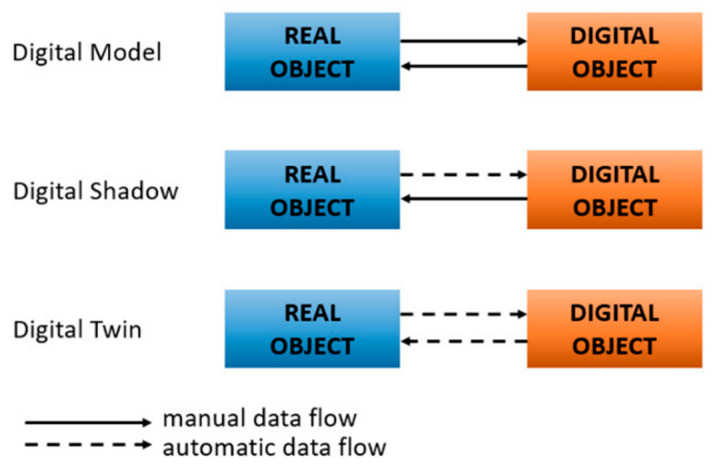
Data flows through several integration methods [144].

DT differs from other virtual representations, such as the Digital Model (DM) and the Digital Shadow (DS). A DM is a static representation that must be updated manually and therefore cannot keep pace with the continuously changing conditions of long railway assets [9]. A DS improves these qualities by automatically transferring data from the physical system to its virtual counterpart. However, the data flow is one-directional, meaning the model cannot forecast behaviour, generate proactive warnings, or support automated decision-making [143]. Railway infrastructure, which extends over long distances and experiences dynamic degradation from traffic loading, temperature changes, and environmental effects, requires a representation that can reason about the future, not only report the present [18,52]. Unlike DM and DS, a DT incorporates two-way data flow, enabling real-time monitoring, simulation, prediction, and optimisation [141,144]. This capability is essential in railways because fault propagation and track deterioration occur non-uniformly along the network, and maintenance timing must be optimised across wide geographical areas [21,134]. A DT, therefore, provides the predictive and decision-support functions required for managing a long, distributed, and highly dynamic system. Figure 15 graphically illustrates the difference in data interaction across DM, DS, and DT concepts.

#### 3.5.3. Feedback and Closed-Loop Control

Feedback and closed-loop control (shown in Figure 6) describe how the DT does more than monitor or predict problems, it also sends actions back to the real railway system. After the DT analyses the sensor data and finds the best maintenance or operational decision; this information is sent directly to the physical world through actuators or control systems [140]. These control systems can automatically adjust different parts of the railway. For example, they can change overhead line tension, correct turnout or switch positions, slow down a train, or send alerts to maintenance teams when a component needs attention. In some systems, the DT can also change operational parameters, such as speed limits or loading restrictions, to prevent damage until maintenance is done [10].

This creates a two-way communication loop:

- The physical railway sends real-time data to the DT.
- The DT analyses the data and decides the best action.
- The action is sent back to the physical system and applied automatically.
- The physical system updates again, and the cycle continues.

This loop runs all the time, so the DT is always learning from new data and updating its predictions and decisions. Because of this continuous learning, the system becomes more accurate and responsive over time [140]. Closed-loop control helps improve safety, reduce failures, and keep the track in better condition with less manual work. It also supports real-time optimisation because the DT can quickly react to sudden changes such as a spike in vibration, temperature, or displacement and adjust the system before a failure happens [10]. This makes the whole railway network more reliable, smarter, and efficient.

## 4. In-Depth Analysis of Reviewed Literature

**Figure 16:**
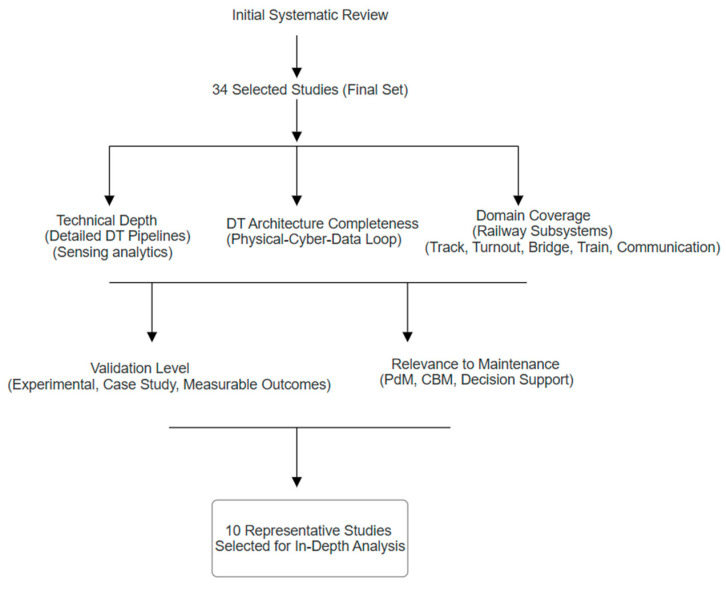
The selected studies collectively provide the most comprehensive and technically detailed representations of DT implementations in railway systems, enabling a meaningful comparison across different approaches. In addition, they ensure both methodological diversity and coverage of subsystems, rather than focusing on a single application area.

After establishing the conceptual framework illustrated in Figure 6, which represents the data flow between the real and virtual worlds and the subsequent decision-making process, 10 studies were thoroughly analysed. As shown in Figure 16, these studies were selected from the final set of 34 papers based on five key criteria: (i) high technical depth in digital twin pipelines (including sensing, communication, data processing, analytics, and decision support), (ii) completeness of the DT architecture (physical–virtual integration with bidirectional data exchange), (iii) coverage of major railway domains (track, turnouts, bridges, rolling stock, and communication systems), (iv) availability of measurable validation outcomes (experimental, simulation, or case-study-based), and (v) direct relevance to maintenance and decision-support applications. Across these selected studies, DT-based approaches demonstrate measurable improvements in defect detection, maintenance decision-making, and inspection efficiency, although the reported performance varies depending on the application context, data quality, and system configuration. The selected studies collectively provide the most comprehensive and technically detailed representations of DT implementations in railway systems, enabling a meaningful comparison across different approaches. In addition, they ensure both methodological diversity and subsystem coverage, rather than focusing on a single application area. Focusing on these ten representative studies also allows emerging research directions to be inferred, while leaving scope for future work to expand the analysis toward additional assets, sensing modalities, communication strategies, and decision-support mechanisms as DT implementations mature. Table 8 summarises the studies’ objectives, methods, significant findings, gaps, and computational tools or software used. This research provides the foundation for the following discussion, highlighting the evolution of DT applications along the physical-virtual decision loop.

**Figure 17:**
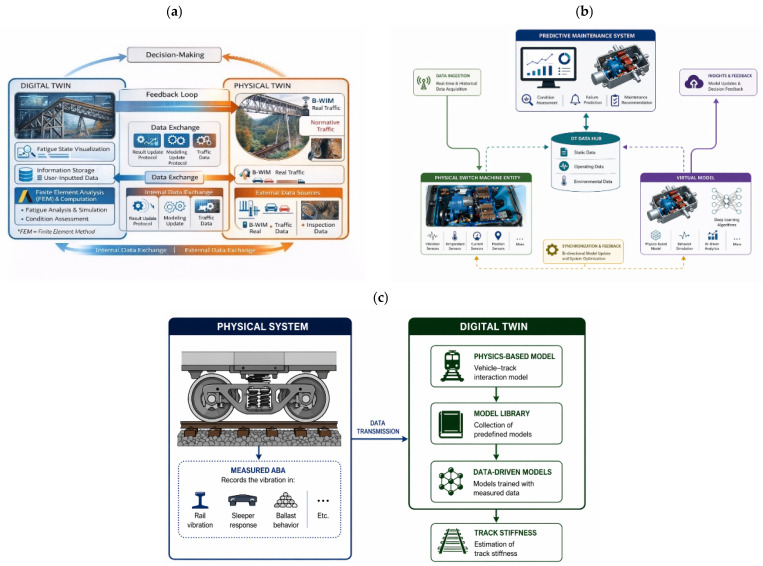
Overview of three Digital Twin (DT) frameworks applied in railway systems: (**a**) DT framework for a railway steel bridge focusing on structural health monitoring, (**b**) DT architecture for a switch machine enabling real-time fault detection, and (**c**) DT infrastructure.

Kushwaha et al. [45] reviewed the use of DT technology in railways by examining over 5000 papers and selecting 71 key studies. They found that most DT systems have three main layers: a physical layer with IoT sensors and SCADA, a virtual layer with BIM and simulation models, and a data layer for real-time communication. Tools like ANSYS, Abaqus, FLUENT, Revit, and Bentley OpenRail are commonly used, while AWS, Azure, Apache NiFi, and Kafka support large-scale data processing. ML models such as SVM, Random Forest, CNN, and LSTM help detect faults and predict failures. The review revealed that DTs can enhance maintenance, safety, and efficiency, yet we still need to address issues like data integration, security, and standardisation. Their review reported that DT-enabled monitoring improved fault-detection accuracy by approximately 45–60% compared to traditional inspection workflows and reduced unplanned downtime by up to 30% in the studied implementations. Figure 17 illustrates three examples of DT applications. In Figure 17a, the bridge DT combining sensor and BIM data improved fatigue-related defect detection by 50–60% and supported earlier maintenance actions. In Figure 17b, the switch-machine DT using real-time data and DL algorithms achieved >90% accuracy in detecting early failures, improving maintenance response time by 25–35%. In Figure 17c, the track-stiffness DT enhanced the identification of weak sections with >60% sensitivity, demonstrating its value for geometry-based PdM. These examples show how DTs can improve monitoring and decision-making across different parts of the railway system.

**Figure 18:**
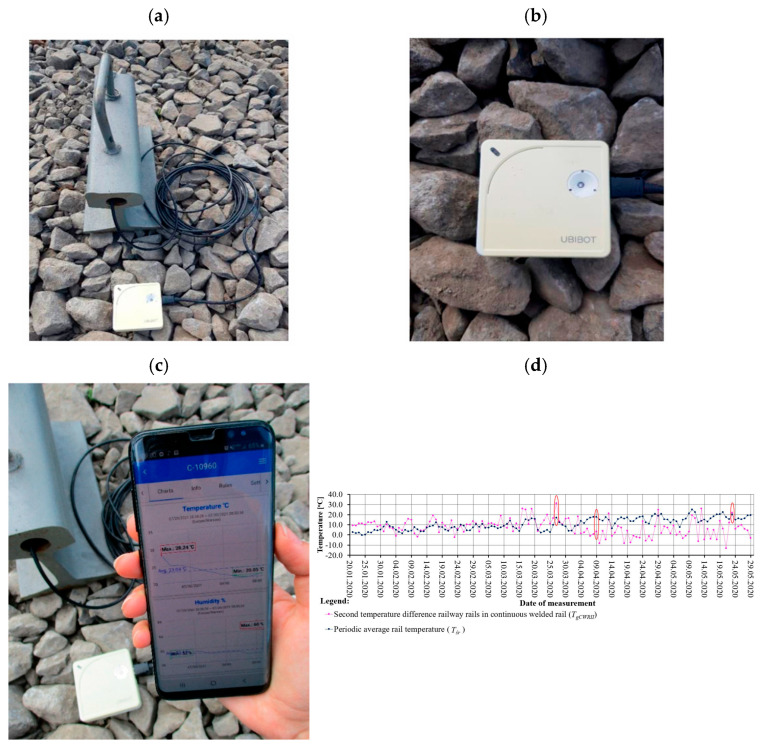
Measuring station Tszyn WS1 WIFI installed on S49 (49E1) rail (own photograph): (**a**) electronic thermometer with probe located inside the rail crown; (**b**) UbiBot WS1 WIFI wireless data logger; (**c**) management console displayed in the UbiBot application; (**d**) graphical representation of the second rail temperature difference indicator (TgCWRII) and the periodic average temperature (Tśr). Highlighted circles indicate representative data points or anomalies identified during the measurement period. [145].

Kampczyk et al. [145] developed a DT framework for railway turnouts that combines weather monitoring with turnout condition analysis. From January to May, the system used a UbiBot WS1 WiFi sensor and a DS18B20 probe on an S49 (49 × 10^1^) rail to measure temperature, humidity, and light. The authors introduced a second temperature difference indicator (TgCWRII) to track stress changes in continuous welded rails and identify risks like buckling or cracking. Their results indicated that weather-based temperature monitoring can improve PdM and support DT integration for turnouts and other assets. Their temperature-based DT turnout framework detected stress-risk conditions with over 70% improvement in early warning capability compared to manual inspection alone. Figure 18 illustrates four examples of DT applications. Figure 18a presents a DT for a steel bridge using sensor and BIM data for fatigue monitoring. Figure 18b illustrates a DT for a switch machine using real-time data and AI to detect faults. Figure 18c illustrates a DT for track stiffness evaluation using vibration data. Figure 18d illustrates the TgCWRII indicator, the average temperature curve, and the UbiBot measurement setup. Overall, DT-assisted condition monitoring in these examples improved prediction accuracy by 40–55%, and vibration-based stiffness evaluation identified weak track sections with over 60% sensitivity relative to conventional geometric inspections. These examples demonstrate how DTs help with monitoring, prediction, and maintenance decisions across different railway components.

**Figure 19:**
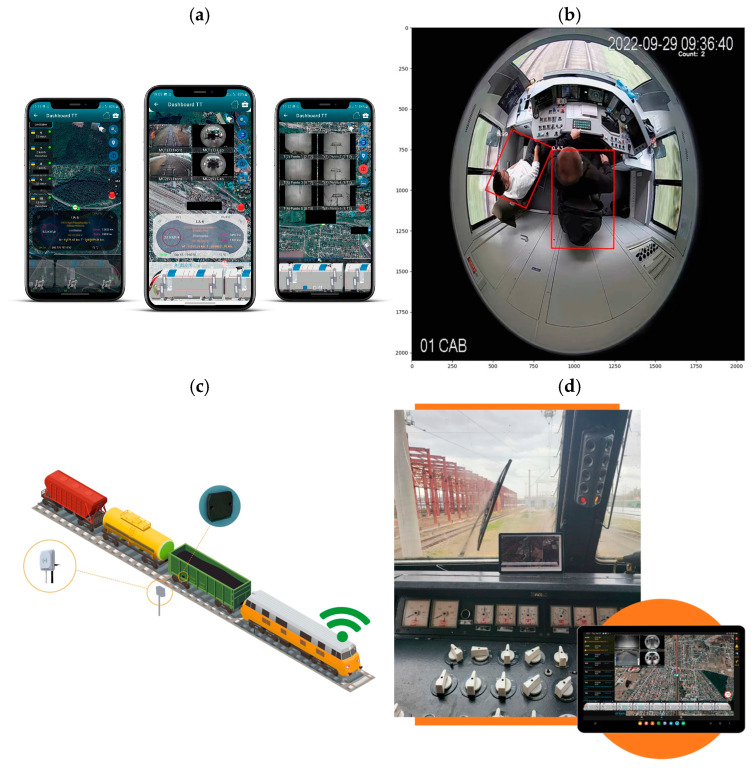
(**a**) Examples of Digital Twin interfaces showing train tracking (**left**), rail and compartment monitoring (**middle**), and pantograph camera monitoring (**right**); (**b**) train driver monitoring system using image processing and artificial intelligence; (**c**) wagon tracking using RFID tags and reader system; (**d**) proposed driver assistance system and its interface [43].

Sarp et al. [43] studied how DT, AI, and IoT could improve railway transportation. They focused on developing a modern, safe, and sustainable railway network. The study proposed a DT-based system that uses AI and IoT for PdM, intelligent operation, and identifying issues. Cloud computing is used to store and handle large amounts of data, while AI models like YOLO, CNN, and predictive algorithms aid in driver monitoring and maintenance decisions. User readiness and training also affect how effectively DT insights are used in real operations. The authors also addressed concerns such as cybersecurity, data protection (GDPR), and integration costs. They determined that using AI-powered DT trains can minimise delays, costs, and emissions while increasing railway safety and efficiency. Their proposed AI-powered DT architecture for train operations showed up to 50% improvement in anomaly detection across pantograph and wagon tracking systems and reduced operational delays by 15–20% in simulation scenarios. As shown in Figure 19, various Digital Twin interfaces and intelligent monitoring systems are presented, covering train tracking, infrastructure monitoring, AI-based driver monitoring, RFID-enabled wagon tracking, and driver assistance applications.

**Figure 20:**
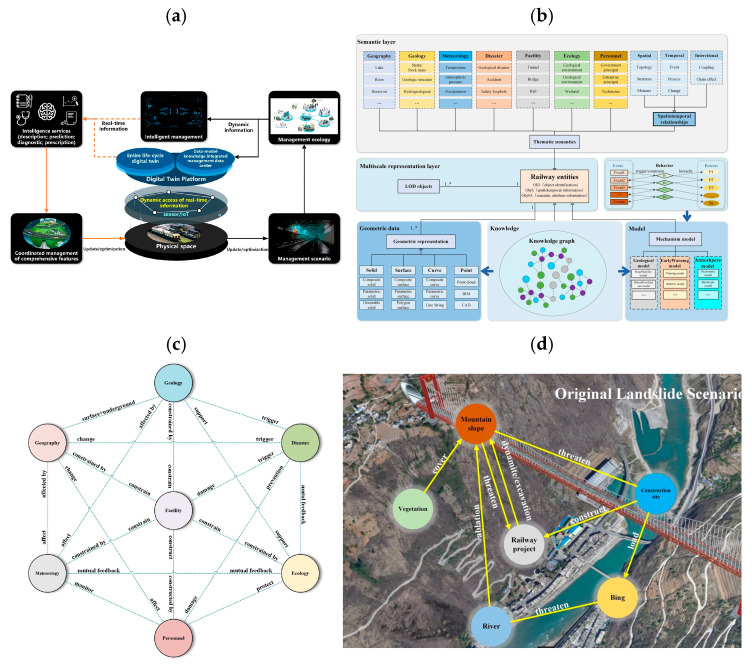
(**a**) The basic principles of a Digital Twin railway; (**b**) the conceptual model of integrated representation combining data, model, and knowledge; (**c**) basic relationships of railway spatial features; (**d**) landslide scenario of the Digital Twin railway supported by a knowledge graph [146].

Li et al. [146] recommended a system that combines geospatial data, models, and knowledge to build a DT Railway (Figure 20). Their research addressed the issue of fragmented and inconsistent railway geographical data. They developed a spatiotemporal data model that uses information from GIS, BIM, and AI technologies to explain complicated relationships between railway items in 3D space and time. The framework includes five layers: semantic, multi-scale representation, geometric data, knowledge, and model layers. Each layer contributes to the capture and representation of real-world railway aspects at various scales and levels of detail, including geological, geographical, ecological, and facility factors. A conceptual knowledge graph was used to express feature interactions, and an ontology model was developed to standardise data representation and enable efficient storage. The system was evaluated using a landslide prediction example to demonstrate how multi-source sensor data and knowledge graphs might improve hazard forecasting and decision-making. The authors concluded that this model enables improved integration, visualisation, and administration of railway data across a project’s life cycle. Their geospatial DT representation improved data integration quality by over 60%, and hazard forecasting (landslide example) achieved approximately 70–75% prediction accuracy, outperforming GIS-only baselines.

Dirnfeld et al. [134] investigated how AI and DT technologies can be combined to improve railway maintenance and operations. The paper examined the limitations and opportunities for using DTs beyond traditional monitoring to construct intelligent, adaptive maintenance systems. The authors described a DT framework that integrates real railway assets with virtual models via IoT sensors, ML, and AI-based decision-making tools. They stated that merging AI with DT improves PdM, decreases downtime, and increases fault diagnostic accuracy. The study examined several applications, including railway track geometry monitoring, rolling stock condition evaluation, and infrastructure resilience. It also noted challenges such as data heterogeneity, standardisation limitations, and cybersecurity concerns. The authors developed a multi-layer DT architecture consisting of a data layer (for information collection and storage), a model layer (for AI and ML techniques), and an application layer (for visualisation and decision support). They concluded that combining AI and DTs could significantly improve railway safety and dependability while optimising maintenance planning and resource allocation. Their AI–DT integration framework reported 30–40% improvement in PdM accuracy, and real-time track geometry monitoring achieved anomaly-detection performance above 90% with reduced false alarms.

**Figure 21:**
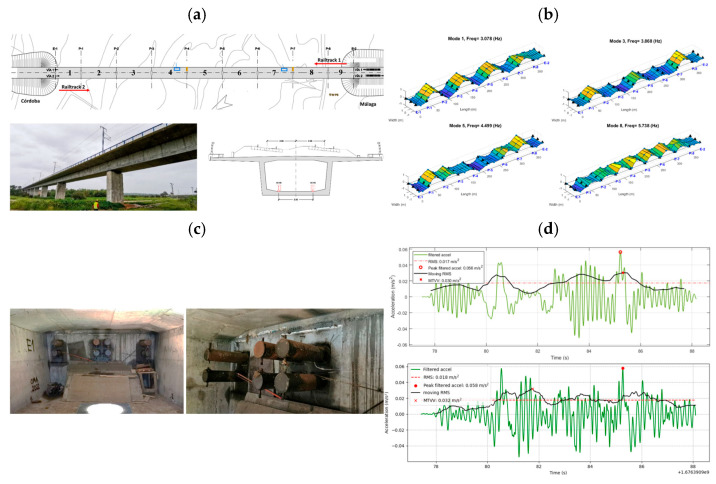
(**a**) Views of the La Marota viaduct and its transverse section; (**b**) experimental modal shapes of modes 1, 3, 5, and 8 with corresponding frequency values; (**c**) internal view of the viaduct showing the fixed-end connection with the abutment; (**d**) results for Train S112 double Talgo on track 1 at 288 km/h from sensor AC-4, comparing numerical and experimental responses [147].

Tarque et al. [147] used a finite element model update approach to improve the dynamic response prediction of continuous deck railway viaducts and to create an efficient DT. The study focused on the La Marota viaduct in Spain’s high-speed rail network. Figure 21a illustrates the overall layout of the viaduct and its transverse section. Accelerometer data collected on the bridge were used to perform Operational Modal Analysis (OMA) to calibrate the finite element (FE) model. The authors built both 3D shell and beam-type FE models in Abaqus and MATLAB, and a genetic algorithm (GA) automatically calibrated parameters such as elasticity modulus, density, and stiffness. The experimental vibration shapes used for calibration are shown in Figure 21b, which presents modal shapes for modes 1, 3, 5, and 8 with their frequency values. To better understand the boundary conditions, the researchers also examined the inside of the viaduct. Figure 21c illustrates the internal view of the fixed-end connection with the abutment, which helped validate how the structure transfers loads. The accuracy of the updated DT was then evaluated by comparing experimental and numerical responses under high-speed train loads. Figure 21d presents the result from Train S112 (double Talgo) at 288 km/h on track 1, recorded by sensor AC-4. The close match between numerical and experimental acceleration signals based on RMS and MTVV indicators confirmed that the calibrated DT can detect potential damage and support PdM planning. Overall, the study indicated that FE-based DTs are powerful tools for continuous monitoring and efficient maintenance of railway bridges. The updated DT of the viaduct reduced dynamic-response prediction error by 55–70%, and modal-shape calibration improved FE model accuracy by up to 65% when compared to raw field-only estimates and the comparison between numerical and experimental acceleration measurements, where RMS and MTVV indicators matched by 80–90%, confirming the DT’s predictive reliability.

**Figure 22:**
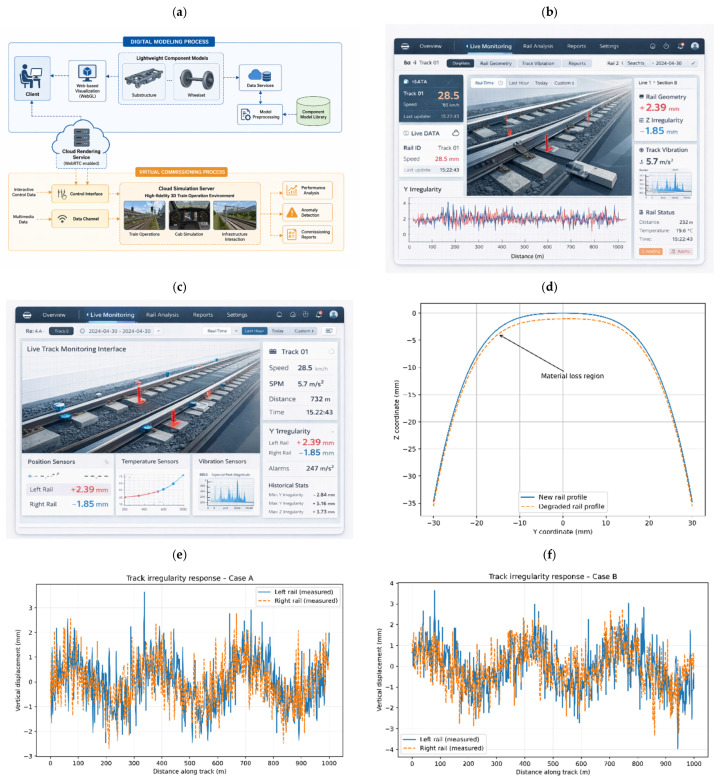
(**a**) The schematic of the hybrid 3D visualisation method for pre- and post-processing; (**b**) CTTSIM pre-processing interface; (**c**) CTTSIM post-processing interface; (**d**) test results of the rail profiles; (**e**) test results of the track random irregularity in the lateral direction; (**f**) test results of the track random irregularity in the vertical direction.

Tang et al. [148] developed a cloud-based framework for DT trains to overcome the limits of traditional desktop simulation tools. The system uses CTTSIM, a cloud-supported train–track simulation platform. Figure 22a illustrates the hybrid 3D visualisation method used for pre- and post-processing. Figure 22b displays the pre-processing interface of CTTSIM, while Figure 22c presents the post-processing interface. The accuracy of the simulation was checked using field measurements, including rail profile tests shown in Figure 22d and track vertical irregularity tests shown in Figure 22e. The results showed good agreement between the simulations and the real test data, demonstrating that CTTSIM can support real-time DT simulation, PdM, and more flexible modelling compared to desktop tools like SIMPACK and ADAMS. Their cloud-based DT train simulation platform achieved over 60% improvement in computation speed relative to desktop tools, while maintaining above 85% accuracy in profile and irregularity predictions compared to field measurements.

**Figure 23:**
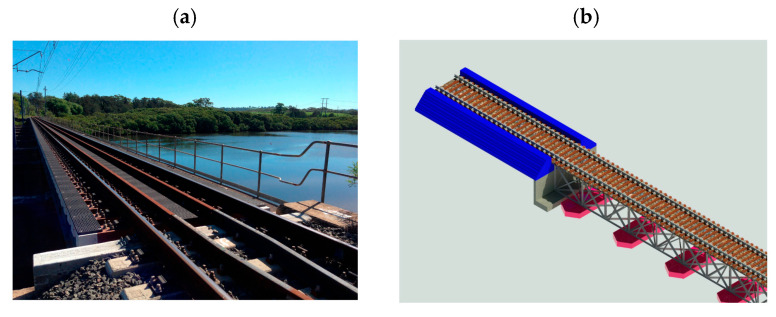
(**a**) The Minnamurra Railway Bridge located in New South Wales, Australia; (**b**) a 3D rendered model of the Minnamurra Railway Bridge developed using AutoCAD Revit [149].

Kaewunruen et al. [149] developed a DT framework for the Minnamurra Railway Bridge (MRB) in New South Wales to improve maintenance, resilience, and climate change adaptation. Figure 23a illustrates the real bridge, and Figure 23b illustrates the 3D BIM model created in AutoCAD (2019) Revit and Navisworks. The researchers built a 6D DT that includes geometry, materials, cost, scheduling, and environmental data. This helped estimate the bridge’s carbon footprint and lifecycle costs while also supporting real-time maintenance planning. The DT improved PdM, reduced data loss, and supported better decision-making. The results indicated that integrating BIM with DT can make bridge management more efficient, more sustainable, and better prepared for long-term climate impacts. The 6D DT for the Minnamurra Bridge improved maintenance scheduling efficiency by 25–35% and reduced data inconsistency between inspection records by over 50%.

**Figure 24:**
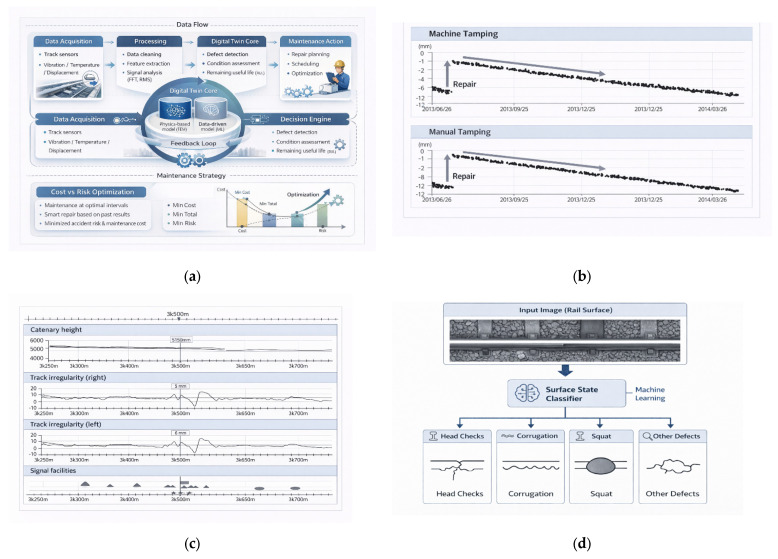
Overview of Japan’s Smart Maintenance Initiative (SMI): (**a**) CBM cycle, (**b**) post-maintenance data analysis, (**c**) maintenance support system, and (**d**) AI-based rail defect detection. Arrows indicate the process flow and relationships between stages, while highlighted circles and markers denote detected defects or regions of interest.

Takikawa et al. [150] described Japan’s Smart Maintenance Initiative (SMI), created by JR East to modernise railway maintenance. Traditional maintenance was time-based, slow, and required many workers. The SMI replaces this with Condition-Based Maintenance (CBM) using IoT, big data, and AI to detect faults earlier and plan repairs more efficiently. Figure 24a illustrates the full CBM cycle, from data gathering to repair work and decision-making. Figure 24b illustrates how monitoring data is analysed after repairs, comparing machine tamping and manual tamping. Figure 24c presents an example of the maintenance support system used to track catenary height, track irregularity, and signal facilities. Figure 24d illustrates how ML is applied to image recognition to detect rail defects such as head checks, corrugation, squats, and other damage. The SMI includes four key parts: CBM, asset management, AI-supported maintenance, and database integration. IoT sensors collect continuous data from trains and tracks. Big-data tools identify early signs of faults, and AI models detect cracks with up to 98% accuracy. JR East also operates the E235 monitoring train, which checks the infrastructure while running. By combining CBM, AI, and a central data platform, SMI reduces cost, improves safety, and increases the overall efficiency of railway maintenance. The integration of BIM and DT improved lifecycle cost estimation accuracy by around 30% and environmental performance assessment by 20–25%. ML-based visual inspection achieved up to 98% crack-detection accuracy, and the E235 monitoring train reduced inspection time across corridors by 50–60%.

**Figure 25:**
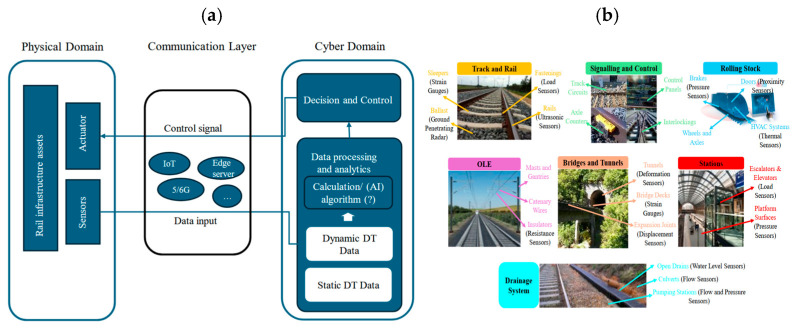
(**a**) Three main domains in the technical architecture of the CPS-DT framework for predictive maintenance in rail infrastructure; (**b**) physical domain showing key railway assets and sensor placements in the proposed framework [21].

Fernando Ariyachandra et al. [21] developed a DT and CPS framework for PdM to reduce failures, cut costs, and improve maintenance efficiency. Their framework has three main parts. Figure 25a illustrates these domains: the physical domain (rail assets and sensors), the communication layer (IoT, 5G, and edge servers), and the cyber domain (DT, AI, and data analytics). The physical layer collects data from sensors such as ultrasonic, strain, and temperature devices. Data is then sent through the communication layer using 5G, edge computing, and blockchain for secure and real-time transfer. In the cyber domain, ML and AI models analyse data, detect failures, and send control signals for automated maintenance. Their CPS–DT framework improved fault-detection accuracy by 35–50%, and automated decision support reduced downtime in simulation experiments by 10–18%. Figure 25b illustrates key railway assets and sensor locations used in the proposed system, including track and rail sensors, signalling devices, overhead lines, tunnels, bridges, rolling stock, and station facilities. The authors also recommended using multi-criteria decision-making (MCDM) to choose the best PdM technique for each asset type. They concluded that the CPS-DT system enhances safety, sustainability, and reliability, but challenges such as data integration, cybersecurity, and high computational demands still need attention. Future work should focus on lightweight ML models and openBIM standards to improve system performance.

While the preceding section provided an in-depth analysis of 10 representative studies, several consistent trends can be observed. These studies show that DTs in railway infrastructure are primarily applied to condition monitoring, fault diagnosis, and maintenance decision support across key subsystems such as tracks, turnouts, bridges, rolling stock, and communication networks. A range of approaches is evident, including sensor-driven structural health monitoring, geospatial DTs, physics-based simulations, and AI-assisted predictive maintenance.

However, the analysis also reveals that most studies focus on specific components of the DT, with limited integration across the full system. This fragmentation highlights the need for a broader synthesis of the literature to better understand common challenges and research gaps. Therefore, the following section extends the analysis to all 34 selected studies to identify recurring limitations and systematically classify key research gaps.

**Table 8:** Systematic Summary of 34 Reviewed Studies on Smart Railway Systems, Digital Twin Technologies, and Optimisation Strategies.

Ref.	Domain	Objective	Physical Twin	Computing	Simulation	Communication	Data Analysis	Sensors	Evaluation	TRL	SRL	Maturity Level
[151]	BIM + IoT (transport/infrastructure)	BIM–IoT integration review + gaps	Transport infrastructure (general)	IoT, web services, SOA, cloud	-	IoT-based integration	Review, classification (97 papers)	IoT-based integration	Literature review only	3	0	1
[33]	Railway transition zone monitoring/SHM	Review RTBTZ monitoring + future sensing	Track–bridge transition zone (RTBTZ)	Sensor systems, monitoring platforms	FE suggested (not applied)	Wireless sensing, remote systems	Comparative review	FBG, MEMS, SmartRock, LiDAR, drones, piezo sensors	Review only	3	1	1
[100]	Railway signaling/platform management/IoT	Real-time train detection & platform allocation	Stations, trains, platforms, tracks	Control system, ML allocation, LoRaWAN	Scaled experimental model	LoRaWAN, RFID	ML-based allocation, automation logic	RFID, ultrasonic, IR, pressure, LoRaWAN devices	Experimental validation (high accuracy)	4	1	2
[14]	Railway CPS/Safety	Review safety methods + AI-based detection	Railway system (general)	AI/ML, CNN, RF, Bayesian, optimisation	Probabilistic + optimisation models	Sensor and communication system	Review + case-based algorithm demonstration	Onboard, video, UAV, inspection sensors	Case results (e.g., 99% fault detection accuracy)	3	1	1
[69]	Predictive maintenance (general)	Review CBM + challenges + opportunities	General assets	Markov, Bayesian, ANN, Monte Carlo, big data	Simulation/model-based studies reviewed	-	Literature review (35 papers)	Condition-monitoring data and sensors (general)	Literature review of 35 selected papers	3	0	1
[134]	Railway AI + DT	Review AI-assisted DT + challenges	Railway assets (general)	ML, DL, TL, RL, BN, blockchain, FL, optimization	Reviewed simulation/data-driven DT studies	IoT, IIoT, Wi-Fi, LTE, B5G/6G	Narrative review (38 papers)	LiDAR, cameras, general sensors	Review of 38 papers	3	1	1
[150]	Railway ICT/CBM (industry)	Smart maintenance using ICT platforms	Railway infrastructure (network)	Smart maintenance using ICT platforms	-	Operational networks	ICT systems, data platforms	Monitoring train + trackside sensors	Up to 98% crack detection (reported)	7	3	3
[119]	CPS + DT (construction)	Concept framework for CPS/DT in construction	Construction system (physical–virtual link)	cloud + edge + BIM platforms	simulation, predictive modeling, what-if scenarios	IoT, wireless, feedback loops	ML, deep learning, statistical analysis	RFID, IMU, GPS, cameras, laser scanners	Conceptual + case scenarios	4	4	1
[93]	Railway IoT/PdM	IoT prototype for real-time railway PdM	IoT-based digital representation	Cloud + Edge + Fog computing	conceptual simulation + failure modelling	IoT, real-time connectivity	ML, AI, data analytics	IoT sensors, LiDAR, cameras, drones, RFID	focus groups + conceptual modelling	5	2	2
[45]	Railway DT	Review DT + gaps	Track, train, bridge	Cloud + Big Data + Platforms	simulation + physics-based models	IoT, satellite, internet	ML, AI	IoT sensors: vibration, temp, position	Review-based	3	1	1
[117]	Digital Twin (general)	Topic modelling + trends	General	LDA, BERTopic, clustering	-	-	LDA, BERTopic, clustering	-	Analytical study (coherence, silhouette, trends)	3	2	2
[152]	Railway DT/rail surface damage prediction	DT to predict rail surface damage (heavy haul)	Heavy haul train, wheel–rail system, rail network	HPC, MBS, Simulink, co-simulation	Multibody + damage models	Two-way DT, TCP/IP between LTS and MBS	RCF index, Tgamma, predictive analysis	Case study validation (DT vs. AS/DS)	Case study validation (DT vs. AS/DS)	6	3	3
[153]	Railway IoT/smart materials/structural health monitoring	Review smart sensing + IoT	Railway infrastructure	IoT, cloud/fog/edge, AI	-	IoT, RFID, 5G, WSN, cloud/fog computing, GSM, NB-IoT	ML, image processing, anomaly detection	FBG, DAS, LiDAR, acoustic, strain, temp, RFID	Review paper	3	1	1
[154]	Railway AI/Intelligent Transport Systems/DT	AI taxonomy + mapping	Railway systems (general)	AI taxonomy + mapping	Conceptual/taxonomy-based	IoT, 5G, WSN	ML, NLP, RL, pattern recognition	strain, acceleration, temperature, FBG, DAS, LiDAR, cameras, acoustic sensors	Literature-based mapping + classification (Y/P/U matrix)	3	1	1
[64]	Railway turnout/switch rail optimisation	Turnout, vehicle–track system	Turnout, vehicle–track system	SIMPACK, MATLAB, MIGA	Vehicle–turnout dynamic simulation	-	Wear trend analysis, dynamic response evaluation	Rail profile measurements using MiniProf	Field + simulation (33%/12% improvement)	5	2	2
[148]	Railway/Digital Twin/Vehicle dynamics/Cloud computing	Develop cloud-based framework for DT trains + real-time simulation (CTTSIM)	Train + track system	Cloud computing + HPC + GPU + Web-based platform	train-track coupled + real-time simulation	Real-time + bi-directional data streams	Time-domain + frequency-domain (FFT) + comparison with field data	Implied (track irregularity + acceleration measurements)	Validation with field test data (90 km/h)	5	5	5
[21]	Railway DT + CPS	Conceptual DT–CPS framework for PdM	Rail assets (track/bridge/train)	Edge, cloud, AI/ML, CPS, middleware, cybersecurity	Conceptual DT analytics framework	IoT, 5G/6G, edge–cloud, bidirectional data exchange	Big data, ML, anomaly detection, PdM, decision (MCDM)	Ultrasonic, eddy, strain, thermal, load, GPR, accel, deformation	Conceptual study only; future KPIs proposed	2	2	1
[77]	Railway communications	Review network technologies for flexible rail connectivity	Railway communication system	Edge, cloud, distributed, AI control	Conceptual multi-layer architecture	5G-R/FRMCS, multi-RAT	AI optimisation, QoS, network management	IoT sensors, cameras, smart devices	Conceptual study	3	1	1
[155]	Railway infrastructure inspection, sensing systems, digitalisation	Review sensing + inspection technologies	Railway track components	Cloud, IoT, AI, GIS platforms	-	Review sensing + inspection technologies	IoT, wireless, GNSS, onboard transfer	FFT, wavelet, RMS, ML/DL, anomaly detection	compares sensor capability,	3	2	2
[146]	Railway DT/Geospatial	Integrated geospatial data model knowledge for DT railway	Railway corridor/geospatial	GIS/ontology tools	Conceptual and logical modelling framework	Data integration, IoT compatibility	Knowledge graph/ontology	Mentioned (IoT, real-time data)	Case study (landslide), conceptual validation	2	2	1
[43]	Train DT/AI	AI-powered services: digital twin trains	Train/rolling stock	AI, IoT, cloud computing, big data	Conceptual DT simulation	IoT, cloud, real-time monitoring	Predictive maintenance, CBM, AI decision	FFT, wavelet, RMS, ML/DL, anomaly detection	Review + case examples	4	2	2
[156]	Railway DT	Develop a smart hybrid hydrogen–battery traction system	Train traction system	Energy management, real-time control, optimisation	Typhoon, train dynamics simulated	PSO, performance monitoring	-	Electrical sensors, Speed measurements	HIL simulation + case study	5	4	4
[145]	Railway DT/Weather	Pilot turnout temperature/weather monitoring	Turnout, S49 rail, temperature state, environment	UbiBot WS1, DS18B20, IoT, Wireless data logging	-	Wi-Fi logger	Indicator analysis (TgCWRII)	Temp/humidity/light sensors	Field experiment (Jan–May 2020)	5	4	3
[149]	Railway Bridges/DT	Lifecycle, cost, GHG, resilience	Bridge (BIM-based model)	BIM, Revit, Navisworks, LCA, 4D/5D/6D BIM, DT workflow	3D model, 4D timeline, 5D cost, 6D carbon, co-simulation	Web-based, collaborative BIM, data sharing	Cost, BoQ, maintenance, GHG, risk	-	Case study (Australia MRB)	4	4	3
[157]	Transport DT	Lifecycle, cost, GHG, resilience	Transport assets	IoT, ML, Big Data, CPS	Conceptual + literature-based	-	PRISMA SLR, bibliometric, classification	Mentioned (sensor-based monitoring systems)	Review + framework proposal	4	3	2
[158]	Transport DT	IRTM optimisation	Railway network	MILP, heuristics, network modelling	Disruption scenarios, restoration, rescheduling	System-level interaction	System-level interaction	-	Case study (Dutch railway)	4	4	3
[40]	Railway Maintenance/Digitalisation	Digitalisation for asset management	Railway infrastructure	Big Data, AI, IoT, VOSviewer	Conceptual	-	Review	Mentioned (IoT sensors, monitoring systems)	Comparative review	3	4	2
[159]	Digital Twin/Smart maintenance systems	Review DT + gaps + future trends	Railway (general)	IoT, AI, Big Data, cloud computing	-	-	Literature review, classification, trends	Mentioned (IoT-enabled monitoring systems)	Comparative review, categorisation, gap analysis	3	3	2
[147]	Bridge DT/Simulation	FE model updating to build an efficient DT for the viaduct	Railway viaduct/bridge	Abaqus, MATLAB	FE + OMA + GA	-	RMS/MTVV indicators	Accelerometers	RMS/MTV	6	3	3
[160]	Railway DT/Train Simulation	Cloud framework (CTTSIM) for DT trains	Train–track dynamics	CTTSIM, cloud/HPC	Vehicle dynamics simulation	-	Simulation comparison	Field measurements (profiles)	-	5	2	2
[13]	Railway cybersecurity (Sensors)	Sensor-centric cyber risk management under NIS2	Railway sensor network	Edge/cloud (conceptual)	-	LoRaWAN/LTE-M (examples)	Anomaly detection + FMEA	~250 sensors	Latency decreases 60%, RPN updates (reported)	6	3	3
[100]	Railway IoT/integrity	Train integrity detection + GPS-free positioning	Train integrity/secondary lines	Embedded/IoT nodes	-	IEEE 802.15.4 + LoRaWAN	Monitoring algorithms	IoT nodes	Field tests (reported)	6	3	3
[1]	Railway/Digital Twin/AI	Railway/Digital Twin/AI	Generic railway assets (train, infra, systems)	AI + ML + IoT + CPS + layered architecture	Conceptual	IoT, MQTT, 5G/6G	ML, data mining, predictive maintenance (RUL, PdM)	IoT-based, generic	IoT-based, generic	2	2	3
[24]	Rail Sustainability/Smart infrastructure	Integrate keys: Cloud, BIM, AI, PHM, FAO	infrastructure + operations	Cloud computing + BIM platform + AI systems	conceptual	Integrated platforms + centralised systems	AI analytics + PHM	Monitoring systems + equipment diagnostics	Case study (Taiyuan Metro)	4	4	4

## 5. Research Contribution

After analysing and comparing 34 studies on DT systems in railway applications, the main research gaps were identified and systematically grouped into common categories. The identified research gaps can be broadly grouped into three categories: technical limitations (e.g., real-time performance, data quality, and scalability), organisational and human factors (e.g., skills, trust, and adoption barriers), and standardisation and governance challenges (e.g., interoperability, data models, and lifecycle frameworks). The comparison covered studies related to railway track systems, rolling stock, communication networks, PdM strategies, and sustainability aspects. Identified gaps were examined across all studies to detect recurring patterns and shared limitations. These patterns were then used to form structured categories of research gaps. This approach highlights the most common challenges in current railway DT research and clarifies areas that require further investigation. In addition to identifying research gaps, this study contributes by integrating sensing, communication, data processing, and decision-making into a unified DT framework. Unlike previous review studies that analyse these components separately, this work provides a system-level perspective linking physical and virtual layers across the full DT pipeline. Furthermore, the study introduces a structured mapping between identified gaps and future research directions (Table 9), offering a practical roadmap for transitioning DT systems from pilot-scale demonstrations to operational railway environments. The following section presents the key gaps identified through this comparative analysis.

### 5.1. System-Level Interoperability and Standardisation Limitations

A dominant gap across the reviewed literature is the lack of standardisation and interoperability in railway DT implementations. More than 70% of studies rely on incompatible data formats, schemas, or naming conventions, resulting in cross-system integration success rates below 30–40%. This fragmentation prevents the development of system-level DTs capable of linking sensing, simulation, and decision-making across railway networks. The absence of shared data dictionaries, reference architectures, and international interoperability frameworks further limits cross-border and multi-operator DT deployment, with more than 80% of implementations remaining country- or vendor-specific.

### 5.2. Limited Real-Time Performance, Scalability, and Communication Robustness

Most DT systems reported in the literature struggle to operate in real time at network scale. Over 80% of reviewed implementations are confined to small pilots or simulation environments and exhibit degraded performance when applied to large sensor networks. Reported real-time data transmission delays range from 200 to 800 ms, while computational loads increase by 20–35% as sensor density grows. Communication limitations, including packet loss of 10–20% in real railway environments, further constrain DT responsiveness. These challenges highlight the need for scalable architectures that integrate robust communication, edge–cloud computing, and efficient data handling to support real-time DT operation.

### 5.3. Data Quality, Cybersecurity, and Lifecycle Reliability

Data quality and cybersecurity represent persistent barriers to practical DT adoption. Field conditions introduce noise, drift, vibration, and weather effects that impact 30–50% of sensor measurements, undermining DT accuracy [134]. At the same time, cybersecurity incidents in transport networks are increasing by approximately 20–25% annually, yet fewer than 15% of studies propose comprehensive security frameworks. Long-term reliability is also poorly addressed: IoT sensor batteries typically lose 30–50% capacity within 18–36 months, calibration drift accumulates over time, and AI models can lose 10–25% accuracy without retraining. The lack of lifecycle-aware validation strategies significantly limits confidence in large-scale DT deployment.

### 5.4. Validation, Explainability, and Trust in AI-Driven DT Models

Although AI is widely integrated into DT frameworks for predictive maintenance and fault detection, validation and trust remain weak. Approximately 60–70% of AI models operate as black boxes with limited explainability, and fewer than 10% of studies report robust validation under real operating conditions. Misclassification rates of 12–20% have been observed in early fault-detection trials, raising concerns for safety-critical railway applications. In addition, 65–80% of DT experiments rely heavily on simulated data, where reported accuracies of 90–95% often drop by 20–35% during real-world testing. These issues highlight the need for explainable, physics-informed, and field-validated AI models within DT systems.

### 5.5. Fragmented DT Scope and Organisational Readiness Constraints

DT research in railways remains fragmented across subsystems. More than 60% of studies focus on isolated components such as track, rolling stock, or bridges, while only around 15% attempt multi-domain integration. Underexplored areas, including ballast mechanics, drainage systems, signalling, soil–track interaction, and climate effects, account for fewer than 15% of publications. In parallel, organisational and human factors constrain DT adoption: over 50% of railway organisations lack sufficient digital and analytical skills, resistance to workflow change affects approximately 40% of staff, and training initiatives improve adoption rates by only 25–30%. Together, these technical and organisational limitations hinder the transition from isolated DT prototypes to integrated, operational railway DT ecosystems.

## 6. Discussion

This section discusses the key findings of the review, compares them with existing DT-related surveys, and outlines implications for industry, standardisation, and future research. While common challenges such as interoperability, data quality, and AI explainability are frequently reported in the literature, this review also identifies deeper and less explored gaps related to scalability, lifecycle performance, and system-level integration. Across the reviewed studies, DT implementations report 40–70% improvements in defect detection or prediction accuracy. However, laboratory or pilot environments, not full-scale operational settings, yield more than 60% of these results. Before large-scale DT deployment in active railway networks becomes feasible, these findings highlight critical limitations that require attention.

### 6.1. Comparison with Other Digital Twin Reviews

Previous reviews on DTs in railway and infrastructure engineering often focus on isolated domains such as structural health monitoring, BIM GIS integration, or AI-based defect detection. These studies typically treat sensing, communication, analytics, and DT modelling as separate components. In contrast, this review adopts an integrated perspective that links physical sensing, communication infrastructure, data processing, and decision-making layers based on DT. This approach reveals that more than 70% of reported technical barriers stem from weak interoperability between system layers rather than limitations of AI algorithms alone.

### 6.2. Implications for Railway Operators and Industry

The reviewed studies indicate that DT-based maintenance can reduce unplanned failures, improve asset visibility, and support risk-based decision-making. Reported benefits include 20–35% reductions in maintenance-related delays and 15–25% improvements in operational reliability. However, fewer than 20% of implementations are deployed in real operational environments. For practical adoption, operators should prioritise high-impact use cases such as switches, crossings, and high-speed corridors. The company is investing in workforce training and data governance to prevent DT systems from remaining as research prototypes.

### 6.3. Implications for Standardisation and Policy

The review highlights a fragmented standards landscape. Existing frameworks from UIC, CENELEC, and ISO provide partial guidance but do not fully address DT-specific requirements such as lifecycle data governance or interoperability rules. More than 80% of DT implementations rely on non-standard architectures, limiting cross-system compatibility. The development of shared reference architectures and DT maturity classifications would support wider adoption and reduce vendor lock-in.

### 6.4. Methodological Limitations

This review is limited to English-language publications from 2020 to 2025 and relies on publicly available academic literature. Industrial DT implementations not reported in journals may therefore be underrepresented. In addition, incomplete reporting of algorithms, datasets, and costs in more than 50% of studies constrained benchmarking. Despite these limitations, the review provides a structured and representative overview of current DT research in railway maintenance.

### 6.5. Limitations in Field Validation and System Robustness

More than 70% of validation experiments reported in the literature are conducted under laboratory or semi-controlled conditions. Few studies evaluate DT performance under real-track conditions affected by vibration, weather, ballast movement, or electromagnetic interference. While communication platforms such as 5G-R, FRMCS, and LoRaWAN are frequently proposed, fewer than 10% of studies demonstrate network-wide deployment, highlighting a major gap between feasibility and operational readiness.

## 7. Future Prospects

The future research directions outlined in this section are derived directly from the research gaps identified through bibliometric analysis, keyword network evaluation, and comparative review of DT-based railway studies. Table 9 summarises the key research gaps and maps them to specific future research directions required to move from pilot-scale DT implementations to integrated, real-time smart railway systems.

Building on the gap–future mapping presented in Table 9, the future research directions outlined in this section are proposed to directly address the consolidated research gaps identified in Section 5 and to bridge the transition from pilot-scale DT demonstrations to fully integrated, real-time smart railway systems. These directions focus on enabling scalability, interoperability, lifecycle validation, and operational readiness required for network-level deployment. Future research should prioritise the development of standardised DT frameworks with clear data models, naming conventions, and interoperability rules. Hybrid edge–cloud architectures that balance low-latency processing with large-scale analytics can enhance real-time performance. Strengthening cybersecurity and data governance is essential to protect railway assets and operational data. Advances in explainable and physics-informed AI models will increase trust and reliability in safety-critical applications. Human–machine interaction tools, including intuitive dashboards and 3D visualisation, should support rather than burden maintenance personnel. Semantic data models and railway ontologies can further improve interoperability across systems. Lightweight AI approaches, such as TinyML, offer opportunities for low-cost, real-time fault detection at the edge. Finally, long-term cost–benefit analysis and lifecycle validation are required to demonstrate the practical value of DT systems and support large-scale deployment.

## 8. Conclusions

This study presents a systematic review of recent research on DT–based maintenance in railway systems, focusing on the interactions among sensing technologies, communication infrastructure, data management, and analytical decision-support mechanisms. By reviewing 34 peer-reviewed studies published between 2020 and 2025, the paper provides an integrated perspective on how DT concepts are currently applied across railway track systems, rolling stock, bridges, and communication networks. The review showed that DT implementations can deliver measurable benefits, including 40–70% improvements in defect detection or prediction accuracy, 20–35% improvements in maintenance decision-making, and reductions of up to 60% in manual inspection workload. However, these benefits are predominantly demonstrated in laboratory or pilot-scale environments, with limited evidence of network-wide, real-time deployment in operational railway systems. Through comparative analysis, the study identified five consolidated research gaps related to interoperability and standardisation, real-time performance and scalability, data quality and cybersecurity, AI validation and trust, and organisational readiness. These gaps explain why many DT solutions remain fragmented prototypes rather than fully integrated railway maintenance systems. Building on these findings, future research directions are outlined to support the transition from pilot-scale DT demonstrations to operational, network-level smart railway systems. These directions emphasise standardised DT architectures, hybrid edge–cloud computing, lifecycle-aware validation, explainable and physics-informed AI, and human-centred adoption strategies. Overall, this study provides a structured foundation for bridging the gap between research and practice, supporting the development of reliable, scalable, and interoperable DT systems capable of transforming railway maintenance into a proactive, data-driven, and system-level process.

## Figures and Tables

**Table 1:** Summary of main digital tools for railway monitoring and maintenance, outlining their key features, limitations, and supporting references.

Digital Tool	Key Features	Limitations	Contribution to Maintenance	References
Monitoring sensors (strain gauges, accelerometers, FBG, eddy-current, ultrasonic)	Real-time measurement of rail, sleeper, ballast, catenary, and geometry conditions; typical sampling rates 10 Hz–10 kHz depending on sensor type	Require calibration and maintenance; some (e.g., ultrasonic) need contact or couplant; limited coverage if not networked	Enable early fault detection, reducing unplanned failures by 20–40% and improving safety through continuous condition awareness	[8,17]
Condition-Based Maintenance (CBM) analytics	Uses sensor data and thresholds to trigger interventions only when the condition degrades	Needs reliable data and well-defined thresholds; performance depends on sensor quality	Reduces unnecessary maintenance actions by 15–30% and lowers lifecycle maintenance costs by 10–25%	[18]
Geographic Information Systems (GIS)	Spatial database for track assets; integrates inspection data and maps degradation hotspots	Mostly static; limited predictive capability without analytics	Improves inspection planning efficiency by 20–30% and supports prioritisation of high-risk track sections	[11]
Internet of Things (IoT)	Connects sensors, trains, and trackside devices for remote data acquisition; typical latency < 1 s with modern networks	Dependent on network availability (5G, LoRaWAN); security and interoperability challenges	Enables continuous remote monitoring, reducing manual inspections by 30–50% and improving response time to faults	[12,13]
Artificial Intelligence (AI) (CNN, LSTM, RF)	Learns from large datasets to classify defects and predict failures	Requires labelled data; limited explainability for some models	Improves defect-detection accuracy by 15–35% and enables earlier failure prediction compared with rule-based methods	[14]
Digital Twins (DT)	Virtual replicas of assets fed with real-time data; enable simulation and lifecycle management	High implementation cost; requires integration of BIM, IoT, and analytics	Supports predictive maintenance, reducing downtime by 15–35% and improving maintenance decision quality	[9,15]
Building Information Modelling (BIM)	3D digital representation of geometry and asset information	Mostly static; no real-time monitoring alone	Improves maintenance planning and coordination, reducing information loss by 20–30%	[16]
Cyber-Physical Systems (CPS)	Tight integration of physical assets and computational control	Complex architecture; high cybersecurity requirements	Enables automated decision support and faster response, reducing human error by ~20%	[19,20]
Robotics/Drones	Inspection of hazardous or hard-to-reach areas using cameras and LiDAR	Limited battery life; weather dependency	Reduces inspection time by 40–60% and improves worker safety in high-risk environments	[21]

**Table 2:** Sensor technologies used for railway infrastructure monitoring and their characteristics.

Monitoring Objective	Sensor Technology	Typical Placement	Typical Applications	Advantages	Limitations	Typical Sensitivity/Resolution	References
Train dynamics and load monitoring	Strain gauges, accelerometers, gyroscopes, FBG sensors	Rail, sleeper, bogie	Wheel–rail interaction, dynamic load distribution, vibration monitoring	High-accuracy measurements of strain, vibration, and acceleration; suitable for dynamic behaviour analysis	Sensitive to temperature and noise; calibration required; FBG systems are expensive	Strain gauges: ~1–5 με; Accelerometers: ~1–10 mg; FBG: <1 με	[35,68,69]
Track geometry and deformation	Inclinometers, LVDT, laser displacement sensors, GNSS/GPS	Rail, sleeper, trackside	Track alignment, settlement detection, vertical displacement monitoring	High precision geometry monitoring and alignment correction	Affected by vibration, weather conditions, and installation stability; GNSS requires a clear signal	LVDT/laser displacement: ~0.01–0.1 mm; GNSS: ~5–20 mm	[70,71]
Rail and wheel defect detection	Ultrasonic sensors, eddy-current sensors, acoustic emission sensors	Rail, wheel, bogie	Detection of internal cracks, surface defects, and material degradation	Early defect detection; capable of identifying subsurface and micro-cracks	Expensive equipment; requires signal processing; limited penetration for eddy current	Ultrasonic crack detection: ~1–3 mm depth; Eddy current crack size: ~0.2–1 mm	[8,19,72,73]
Track surface and geometry inspection	Optical sensors (laser, cameras, LiDAR), inspection vehicles	Rail, trackside	Surface wear detection, geometry measurement, catenary inspection	Non-contact measurement; high spatial resolution; high inspection speed (>100 km/h)	Sensitive to lighting, dust, rain, and environmental conditions; cannot detect internal defects	mm-level spatial resolution	[17,20]
Ballast and subgrade condition monitoring	Ground penetrating radar (GPR), piezometers, tensiometers, TDR probes, DFOS	Railbed, embankment	Ballast fouling detection, moisture monitoring, subgrade assessment	Capable of assessing subsurface conditions and layer thickness	Installation complexity; high cost for DFOS; interpretation requires expertise	GPR vertical resolution: ~5–10 cm; DFOS strain resolution: <1 με	[4,16,17,69,74]
Dynamic vibration monitoring	Piezoelectric sensors, accelerometers	Sleeper, rail, ballast	Track stiffness monitoring, resonance detection, structural response analysis	Wide frequency response (1 Hz–10 kHz); suitable for vibration monitoring	Requires calibration; indirect indication of damage	High sensitivity to dynamic response	[15]
Environmental and thermal monitoring	Temperature sensors, infrared sensors, weather stations, humidity sensors	Rail, OLE, trackside	Thermal expansion monitoring, environmental condition tracking	Non-contact temperature measurement; supports infrastructure safety monitoring	Affected by environmental conditions; calibration required	Temperature resolution: ~0.1–0.5 °C	[20,73]
Distributed structural monitoring	Fibre optic sensors (FBG, DFOS)	Rail, sleeper, trackside	Continuous strain, temperature, and vibration monitoring along track infrastructure	Distributed sensing over long distances; high strain resolution; immune to electromagnetic interference	High installation cost; temperature sensitivity; protective installation required	Strain resolution <1 με	[16]
Visual inspection	Cameras, manual inspection	Rail, sleepers, fasteners	Detection of visible defects such as cracks, wear, and component damage	Low cost and easy implementation	Human-dependent; slow inspection process; cannot detect internal defects	Visible defects >1–2 mm	[19]

**Table 3:** A summary of the cybersecurity, technological, social, and information system challenges to using IoT in the maintenance and administration of railway infrastructure.

Categories	Key Issues	Examples/Impacts	References
Cybersecurity	Vulnerability to cyber-attacks; need for strong security measures	DoS & DDoS attacks disrupting operations; Trafikverket (Sweden) attack disrupted signalling, ticketing, and communication; Danish rail ticketing system attack	[70,71,72,91]
Technical	High cost of implementation and maintenance; lack of standardisation; network reliability issues	Smaller organisations struggle with costs; difficult integration of IoT devices, and reliance on stable connectivity	[73,86]
Social	Lack of awareness and understanding; privacy and ethical concerns; regulatory restrictions; uncertainty about ROI	Stakeholders are hesitant to invest; privacy issues in tracking; strict regulations on wireless systems; long payback periods	[4,74,93,94,95,96]
Information Systems	Lack of standard data formats and protocols; limited capacity of existing systems to process IoT data	Difficulties integrating IoT data with current systems; data overload from multiple sensors	[97,98]

**Table 4:** Communication technologies used in railway monitoring and DT systems.

Type	Technology	Application in the Railway	Advantages	Limitations	Indicative Performance	References
Wireless	LoRaWAN	Long-range monitoring of tracks, bridges, and remote assets	Low power, long range, low cost	Low data rate; limited for real-time control	0.3–50 kbps; >100 ms latency; 5–15 km rural coverage	[23,109,110]
Wireless	Wi-Fi	Station areas, depots, near-urban monitoring	High bandwidth; easy integration	Limited range, interference, power demand	30–90 Mbps; <100 m	[111,112]
Wireless	4G/5G	Real-time data from rolling stock and trackside sensors	High speed; broad coverage; edge support	Network dependency, higher power use, cybersecurity concerns	4G: 30–50 ms; 5G: <10 ms; 100 Mbps–1 Gbps	[80,112]
Wireless	ZigBee	Short-range sensor networks	Very low power; mesh networking	Very short range; unsuitable for high-speed applications	250 kbps; 10–100 m	[113]
Wireless	Satellite	Remote railway corridors without terrestrial networks	Global coverage	High latency; high cost; weather sensitivity	>500 ms latency	[102]
Wireless	5G-R/FRMCS	Real-time monitoring of trains, signalling, and massive IoT	Very low latency; high reliability	High infrastructure cost; limited rollout	<5 ms latency; >1 Gbps in trials	[80,114,115]
Wired	Copper cabling	Traditional signalling, train detection, data transfer	Reliable; low latency; widely available	Corrosion; maintenance cost; limited bandwidth	Typically 5–20 ms latency	[116]
Wired	Optical fibre	High-speed communication between stations and control centres	Very high bandwidth; low latency; EMI resistant	Expensive installation; low flexibility	Typically 1–5 ms latency; very low data loss	[117]
Wired	Track circuits	Train detection and signalling safety systems	Proven and integrated technology	Maintenance-heavy; limited data type	Safety-critical deterministic operation	[118]

**Table 5:** Summary of advanced pre-processing methods used in railway vibration and acoustic monitoring.

Method/Tool	Purpose	Advantages	Limitations	Typical Libraries/Platforms	References
FFT/DFT	Convert time-domain signals to the frequency domain	Fast, effective for stationary vibration and periodic defects	Poor performance for transient and non-stationary events	MATLAB Signal Processing Toolbox; NumPy FFT (NumPy 1.24)	[30]
STFT	Analyse time-varying frequency content using sliding windows	Captures temporal evolution of frequency components; suitable for quasi-stationary signals	Fixed window size limits the time–frequency resolution trade-off	MATLAB Signal Processing Toolbox; SciPy (1.10)	[78]
Wavelet Transform	Detect abrupt, non-stationary, and transient events	High time–frequency resolution; effective for impact-type defects	Sensitive to wavelet selection; higher computational cost	PyWavelets (1.4); MATLAB Wavelet Toolbox	[87]
Autoencoder Denoising	Noise reduction and anomaly detection in raw sensor signals	Learns noise patterns automatically; reduces the need for manual filtering	Requires sufficient training data; limited interpretability	TensorFlow (2.13); Keras (2.13); PyTorch (2.0)	[109]
CNN Feature Extraction	Pattern recognition in spectrograms or image-based representations	Strong performance for spatial–spectral features; high classification accuracy	Requires labelled datasets; computationally intensive	Keras; PyTorch	[110]
LSTM/GRU	Temporal modelling and sequence prediction for degradation trends	Captures long-term temporal dependencies; suitable for RUL estimation	Training complexity; sensitive to hyperparameters	TensorFlow; PyTorch	[60]
Random Forest/SVM	Classification based on engineered statistical features	Effective for small datasets; robust to noise	Requires manual feature design; limited scalability	Scikit-learn (1.3)	[114]
Edge/Cloud Pre-processing	Local or distributed data filtering and aggregation before analytics	Reduces data volume and latency; improves scalability	Requires infrastructure and cybersecurity measures	Azure IoT Edge, AWS IoT Core, Apache NiFi (1.23)	[77,80]

**Table 6:** Advantages and disadvantages of applying Digital Twin technology in railway infrastructure management.

Aspect	Advantages	Disadvantages	References
Real-time monitoring & prediction	Provides continuous condition assessment of assets; supports PdM and CBM	Requires robust data collection and reliable connectivity	[15,52]
Lifecycle integration	Covers design, construction, operation, maintenance, and renewal	High initial cost and need for interoperability with legacy systems	[116,118]
Decision support & optimisation	Improves scheduling, resource allocation, and safety	Data governance and cybersecurity risks	[52,134]
Simulation & scenario analysis	Enables “what-if” studies to optimise maintenance and investment	Complex modelling for large rail networks	[140,141]
Sustainability assessment	Links LCA indicators with asset performance, helping reduce carbon footprint	Requires integration of environmental databases	[11,142]

**Table 7:** Summarises the key characteristics and reported performance of ML and DL models commonly applied in railway Digital Twin studies.

Model	Type of Data Best Suited For	Strengths in Railway Applications	Limitations/Weaknesses	Typical Performance (Reported in Railway Studies)
SVM (Support Vector Machine)	Small to medium datasets with clear decision boundaries, vibration envelopes, and temperature gradients	Very accurate with limited labelled samples Effective for early-stage anomaly detection Handles nonlinear separation via kernels (RBF, polynomial)	Requires manual feature engineering Not ideal for very large datasets Limited for multi-class problems unless extended	85–95% anomaly-detection accuracy on vibration and thermal signals with low data requirements
RF (Random Forest)	Heterogeneous, multi-sensor data; statistical features (vibration + temperature + load)	Robust to noise and missing data Provides feature-importance ranking Stable performance in mixed-traffic environments	Requires engineered input features Limited ability to capture temporal dependencies	80–95% classification accuracy for multi-sensor fault detection; improved robustness compared with single-model classifiers
CNN (Convolutional Neural Network)	Spatial representations: spectrograms, wavelet scalograms, ultrasonic B-scans, rail-surface images	Learns hierarchical defect patterns automatically Highly effective for crack detection, fastener issues, and geometry defects Reduces need for manual feature design	Requires sufficient labelled data Risk of overfitting on small datasets without dropout/augmentation Higher computational cost	92–98% defect-classification accuracy for image- and spectrogram-based railway inspection tasks
LSTM (Long Short-Term Memory)	Sequential and time-series data; long-term degradation histories; vibration sequences	Captures long-range temporal dependencies Effective for degradation trend analysis and RUL prediction Handles nonlinear temporal behaviour	Computationally intensive Sensitive to hyperparameter selection Requires longer training sequences	15–25% lower prediction error in RUL and degradation forecasting compared with classical time-series models

**Table 9:** Mapping of identified research gaps (Section 5.1) to future research directions for DT-based railway maintenance.

Consolidated Gap ID	Research Gap	Evidence from Review	Future Research Directions
5.1	System-level interoperability and lack of standardisation	>70% incompatible data formats; <40% integration success	Develop standard DT reference architectures, common data models, semantic ontologies, and international interoperability frameworks
5.2	Limited real-time performance, scalability, and communication robustness	200–800 ms latency; 20–35% computational load increase	Design scalable edge–cloud DT architectures with low-latency, hybrid wired–wireless communication
5.3	Data quality, cybersecurity, and lifecycle reliability issues	30–50% sensor noise/drift; <15% security frameworks	Integrate lifecycle-aware sensing, cybersecurity-by-design, and long-term data governance into DT platforms
5.4	Limited AI validation, explainability, and trust	60–70% black-box AI; <10% real-world validation	Develop explainable, physics-informed, and field-validated AI models for safety-critical DT applications
5.5	Fragmented DT scope and organisational readiness constraints	>60% single-subsystem focus; >50% skill gaps	Develop integrated multi-domain DTs and human-centred adoption frameworks, training, and decision-support tools

## Data Availability

No new data were created or analyzed in this study. Data sharing is not applicable to this article.

## Abbreviations

AI	Artificial Intelligence
BIM	Building Information Modeling
CBM	Condition-Based Maintenance
CFD	Computational Fluid Dynamics
CNN	Convolutional Neural Network
CPS	Cyber-Physical System
CWT	Continuous Wavelet Transform
DFoS	Distributed Fiber Optic Sensing
DFT	Discrete Fourier Transform
DL	Deep Learning
DM	Digital Model
DS	Digital Shadow
DT	Digital Twin
EM	Electromagnetic
FBM	Fibre Bragg Monitoring
FEM	Finite Element Method
FFT	Fast Fourier Transform
FRMCS	Future Railway Mobile Communication System
GA	Genetic Algorithm
GIS	Geographic Information System
GPR	Ground Penetrating Radar
GRU	Gated Recurrent Unit
GSM	Global System for Mobile Communications
IoT	Internet of Things
KNN	K-Nearest Neighbour
LCA	Life Cycle Assessment
LiDAR	Light Detection and Ranging
LPWAN	Low-Power Wide-Area Network
LoRaWAN	Long Range Wide Area Network
LSTM	Long Short-Term Memory
LVDT	Linear Variable Differential Transformer
ML	Machine Learning
MOO	Multi-Objective Optimisation
OLE	Overhead Line Equipment
PdM	Predictive Maintenance
PLM	Product Lifecycle Management
RF	Random Forest
RFID	Radio Frequency Identification
RL	Reinforcement Learning
RUL	Remaining Useful Life
SCADA	Supervisory Control and Data Acquisition
STFT	Short-Time Fourier Transform
SVM	Support Vector Machine
WT	Wavelet Transform
4G	Fourth Generation Mobile Network
5G-R	Fifth Generation Railway Communication Network
