# Correction: Kazemi et al. Toward Smart Railway Infrastructure Predictive and Optimised Maintenance Through Digital Twin (DT) System. *Sensors* 2026, *26*, 2333

**DOI:** 10.3390/s26113430

**Published:** 2026-05-28

**Authors:** Mahyar Jafar Kazemi, Maria Rashidi, Won-Hee Kang, Mohammad Siahkouhi

**Affiliations:** Centre for Infrastructure Engineering, Western Sydney University, Sydney, NSW 2751, Australiaw.kang@westernsydney.edu.au (W.-H.K.);

## Figure Correction

In the original publication [1], there were mistakes in Figures 1, 6, 17, 22 and 24 as published. The corrected Figure 1, Figure 6, Figure 17, Figure 22 and Figure 24 appear below.

The authors state that the scientific conclusions are unaffected. This correction was approved by the Academic Editor. The original publication has also been updated.

## Figures and Tables

**Figure 1:**
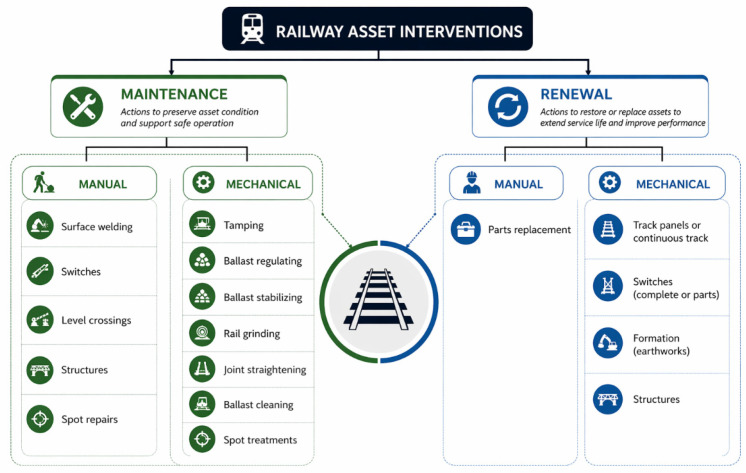
Schematic survey of the maintenance and renewal process.

**Figure 6:**
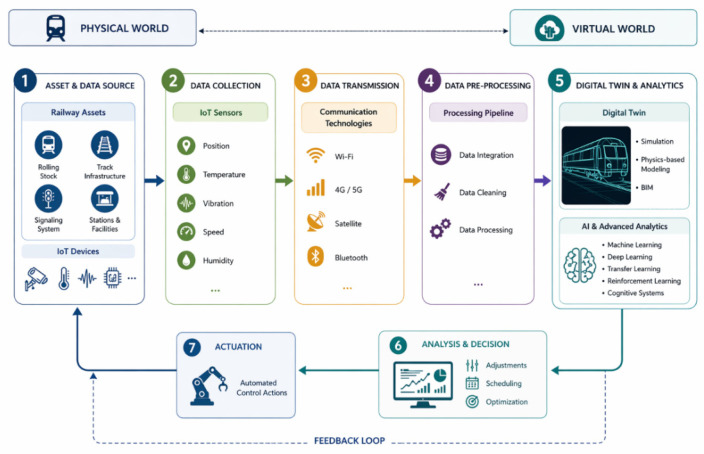
Digital twin for railway.

**Figure 17:**
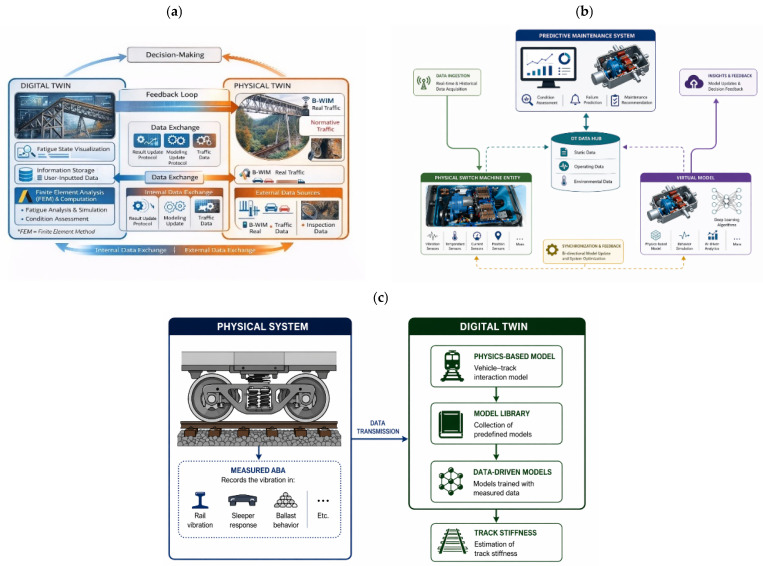
Overview of three Digital Twin (DT) frameworks applied in railway systems: (**a**) DT framework for a railway steel bridge focusing on structural health monitoring, (**b**) DT architecture for a switch machine enabling real-time fault detection, and (**c**) DT infrastructure.

**Figure 22:**
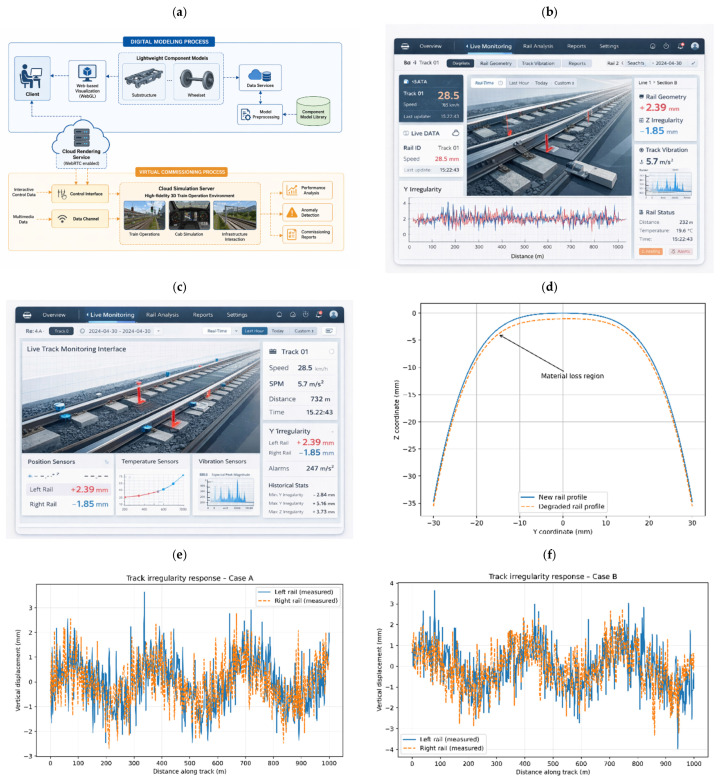
(**a**) The schematic of the hybrid 3D visualisation method for pre- and post-processing; (**b**) CTTSIM pre-processing interface; (**c**) CTTSIM post-processing interface; (**d**) test results of the rail profiles; (**e**) test results of the track random irregularity in the lateral direction; (**f**) test results of the track random irregularity in the vertical direction.

**Figure 24:**
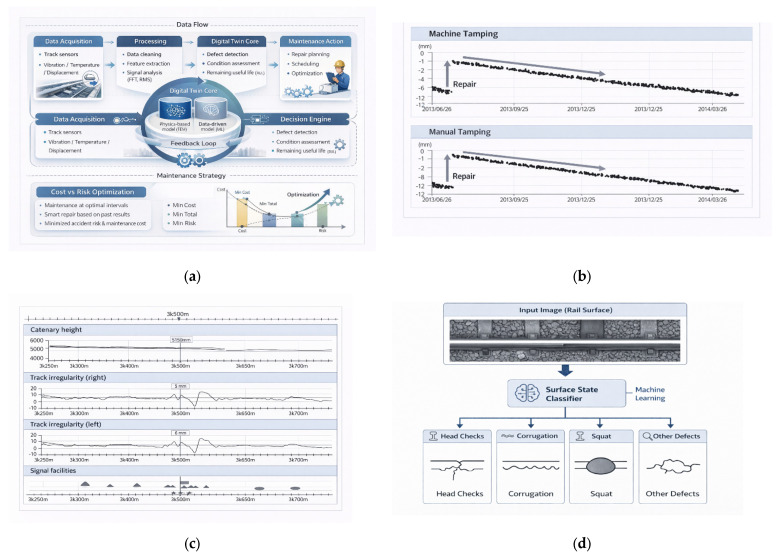
Overview of Japan’s Smart Maintenance Initiative (SMI): (**a**) CBM cycle, (**b**) post-maintenance data analysis, (**c**) maintenance support system, and (**d**) AI-based rail defect detection. Arrows indicate the process flow and relationships between stages, while highlighted circles and markers denote detected defects or regions of interest.
